# A genetic model of congenital intestinal atresia implicates Mypt1 in epithelial organisation

**DOI:** 10.1242/dmm.052605

**Published:** 2026-03-10

**Authors:** Daisuke Kobayashi, Akihiro Urasaki, Tetsuaki Kimura, Satoshi Ansai, Kazuhiko Matsuo, Hayato Yokoi, Shigeo Takashima, Tadao Kitagawa, Takahiro Kage, Takanori Narita, Tomoko Jindo, Masato Kinoshita, Kiyoshi Naruse, Yoshiro Nakajima, Masaki Shigeta, Shinichiro Sakaki, Satoshi Inoue, Rie Saba, Kei Yamada, Takahiko Yokoyama, Yuji Ishikawa, Kazuo Araki, Yumiko Saga, Hiroyuki Takeda, Kenta Yashiro

**Affiliations:** ^1^ Department of Anatomy and Developmental Biology, Kyoto Prefectural University of Medicine, 465 Kajii-cho, Kamigyo-ku, Kyoto 602-8566, Japan; ^2^ Medical Genome Center, Research Institute, National Center for Geriatrics and Gerontology, Obu, Aichi 474-8511, Japan; ^3^ Ushimado Marine Institute, Okayama University, Okayama 701-4303, Japan; ^4^ Graduate School of Agricultural Science, Tohoku University, Sendai 980-8572, Japan; ^5^ Institute for Glyco-core Research (iGCORE)/Life Science Research Centre, Gifu University, 1-1 Yanagido, Gifu 501-1193, Japan; ^6^ Program in Environmental Management, Graduate School of Agriculture, Kindai University, 3327-204 Nakamachi, Nara, Nara 631-8505, Japan; ^7^ Department of Biological Sciences, Graduate School of Science, The University of Tokyo, Bunkyo-ku, Hongo, Tokyo, 113-0033, Japan; ^8^ Laboratory of Molecular Biology, Department of Veterinary Medicine, College of Bioresource Sciences, Nihon University, 1866 Kameino, Fujisawa, Kanagawa 252-0880, Japan; ^9^ Department of Applied Biosciences, Graduate School of Agriculture, Kyoto University, Kyoto 606-8502, Japan; ^10^ Laboratory of Bioresources, National Institute for Basic Biology, 38 Nishigonaka, Myodaiji, Okazaki 444-8585, Aichi, Japan; ^11^ Department of Radiology, Kyoto Prefectural University of Medicine, 465 Kajii-cho, Kamigyo-ku, Kyoto 602-8566, Japan; ^12^ Research Centre for Radiation Protection, National Institute of Radiological Sciences, 4-9-1 Anagawa, Inage-ku, Chiba 263-8555, Japan; ^13^ Research Center for Aquatic Breeding, National Research Institute of Aquaculture, Fisheries Research Agency, 224 Hiruda, Tamaki-cho, Watarai, Mie 519-0423, Japan; ^14^ Department of Gene Function and Phenomics, Mammalian Development Laboratory, National Institute of Genetics, Mishima 411-8540, Japan; ^15^ Department of Genetics, The Graduate University for Advanced Studies, SOKENDAI, Mishima 411-8540, Japan

**Keywords:** Intestinal atresia, Mypt1, Disease model, Actomyosin regulation, Intestinal development

## Abstract

Congenital intestinal atresia (IA) is a birth defect characterised by the absence or closure of part of the intestine. Although genetic factors are implicated, mechanistic understanding has been hindered by the lack of suitable animal models. Here, we describe a medaka (*Oryzias latipes*) mutant, generated by N-ethyl-N-nitrosourea (ENU) mutagenesis, that develops IA during embryogenesis. Positional cloning identified a nonsense mutation in *mypt1*, encoding myosin phosphatase target subunit 1. Mutant embryos exhibited ectopic accumulation of F-actin and phosphorylated myosin regulatory light chain (Mrlc) in the intestinal epithelium, consistent with disrupted actomyosin regulation. These cytoskeletal abnormalities were accompanied by epithelial disorganisation, without notable alterations in cell proliferation, motility or apoptosis. Inhibition of *myh11a*, encoding smooth muscle (SM) myosin heavy chain, ameliorated the IA phenotype, whereas blebbistatin treatment completely rescued the defect, suggesting a non-contractile role prior to SM maturation. Together, these findings demonstrate that *mypt1* loss disrupts intestinal morphogenesis through actomyosin dysregulation. Given the recent clinical identification of IA associated with MYPT1 variants, this medaka model offers a valuable platform to investigate the developmental and molecular basis of *MYPT1*-associated IA in humans.

## Teaser

**Summary:** Loss of *mypt1* causes intestinal atresia in medaka through localised actomyosin dysregulation, establishing a new vertebrate model of MYPT1-associated disease.

## INTRODUCTION

Intestinal atresia (IA) is a congenital defect characterised by complete occlusion of the intestinal lumen, with an estimated incidence of 1.3 to 2.9 per 10,000 live births (Frischer and Azizkhan, 2012). Affected neonates require immediate surgical intervention to restore gastrointestinal continuity. Historically, IA has been attributed to *in utero* vascular accidents that impair blood supply to the developing gut (Louw, 1959; Louw and Barnard, 1955). However, familial clustering and increased prevalence in individuals with trisomy 21 strongly suggest a genetic contribution to disease aetiology (Celli, 2014; Gupta et al., 2013; Louw, 1959; Shorter et al., 2006).

Mouse models support this genetic hypothesis. Targeted deletion of *Fgfr2IIIb* (also known as *Fgfr2b*) or its ligand *Fgf10* results in IA in the absence of mesenteric vascular occlusion (Fairbanks et al., 2004a,b, 2005; Kanard et al., 2005). More recently, variants of *PPP1R12A*, which encodes myosin phosphatase target subunit 1 (MYPT1), have been identified in humans patients with IA and other developmental anomalies, including holoprosencephaly, urogenital malformations and persistent Müllerian duct syndrome (PMDS) (Hughes et al., 2020; Picard et al., 2022). Despite these insights, mechanistic studies of MYPT1-related IA remain limited, as conventional *Mypt1* knockout mice exhibit early embryonic lethality (Okamoto et al., 2005). Actomyosin dynamics play a vital role in morphogenesis (Munjal and Lecuit, 2014). Non-muscle myosin II (NMII)-mediated contractility regulates cell shape, adhesion, migration and tissue organisation (Agarwal and Zaidel-Bar, 2018; Munjal and Lecuit, 2014; Munjal et al., 2015; Vasquez et al., 2014). Phosphorylation of the myosin regulatory light chain (Mrlc) promotes contraction (Vicente-Manzanares et al., 2009), while myosin light chain phosphatase (MLCP) complex dephosphorylates Mrlc to induce relaxation. MLCP comprises a catalytic subunit Pp1cδ, Mypt1 and a small regulatory subunit, M20 (Ito et al., 2004). Mypt1 enhances MLCP activity and substrate specificity by modulating the catalytic domain conformation (Grassie et al., 2011; Hartshorne et al., 2004). Mypt1 is essential for various developmental processes, including gastrulation, axon guidance, vascular remodelling, and morphogenesis of the liver, pancreas and central nervous system (Angulo-Urarte et al., 2018; Barresi et al., 2010; Bremer and Granato, 2016; Dong et al., 2019; Gutzman and Sive, 2010; Huang et al., 2008; Hughes et al., 2020; Kim et al., 2011; Okamoto et al., 2005; Weiser et al., 2009).

In medaka (*Oryzias latipes*), intestinal morphogenesis begins at stage (st.) 21 with medial migration of the endodermal epithelial monolayer to the ventral midline (Kobayashi et al., 2006). This monolayer then forms a bilayer of mediolaterally elongated cells. In the anterior region, endodermal cells stack and converge at the dorsal midline to form a radial endodermal rod – the intestinal anlage. In contrast, intermediate and posterior gut regions undergo dorsoventral elongation and subsequent cavitation to form a tubular gut. Tube formation proceeds in an anterior-to-posterior sequence, with the anterior and intermediate regions becoming lumenised by st. 25, and the posterior region completing this process by st. 26. During this period, buds of accessory organs, such as the liver and swim bladder, also emerge (Kobayashi et al., 2006).

In this study, we report a novel medaka mutant identified through N-ethyl-N-nitrosourea (ENU) mutagenesis that develops IA during embryogenesis. Positional cloning identified a nonsense mutation in *mypt1*. Despite normal endodermal migration and gut anlage formation, mutant embryos failed to maintain epithelial continuity in the intermediate intestinal region immediately following lumen formation. This disruption coincided with localised accumulation of phosphorylated (p)Mrlc and F-actin, consistent with enhanced actomyosin contractility resulting from loss of Mypt1 function. Our findings establish a genetically defined vertebrate model of Mypt1-associated IA and provide new mechanistic insights into how dysregulated cytoskeletal dynamics compromise epithelial integrity during gut development.

## RESULTS

### The *g1-4* medaka mutant develops IA

**Fig. 1.**
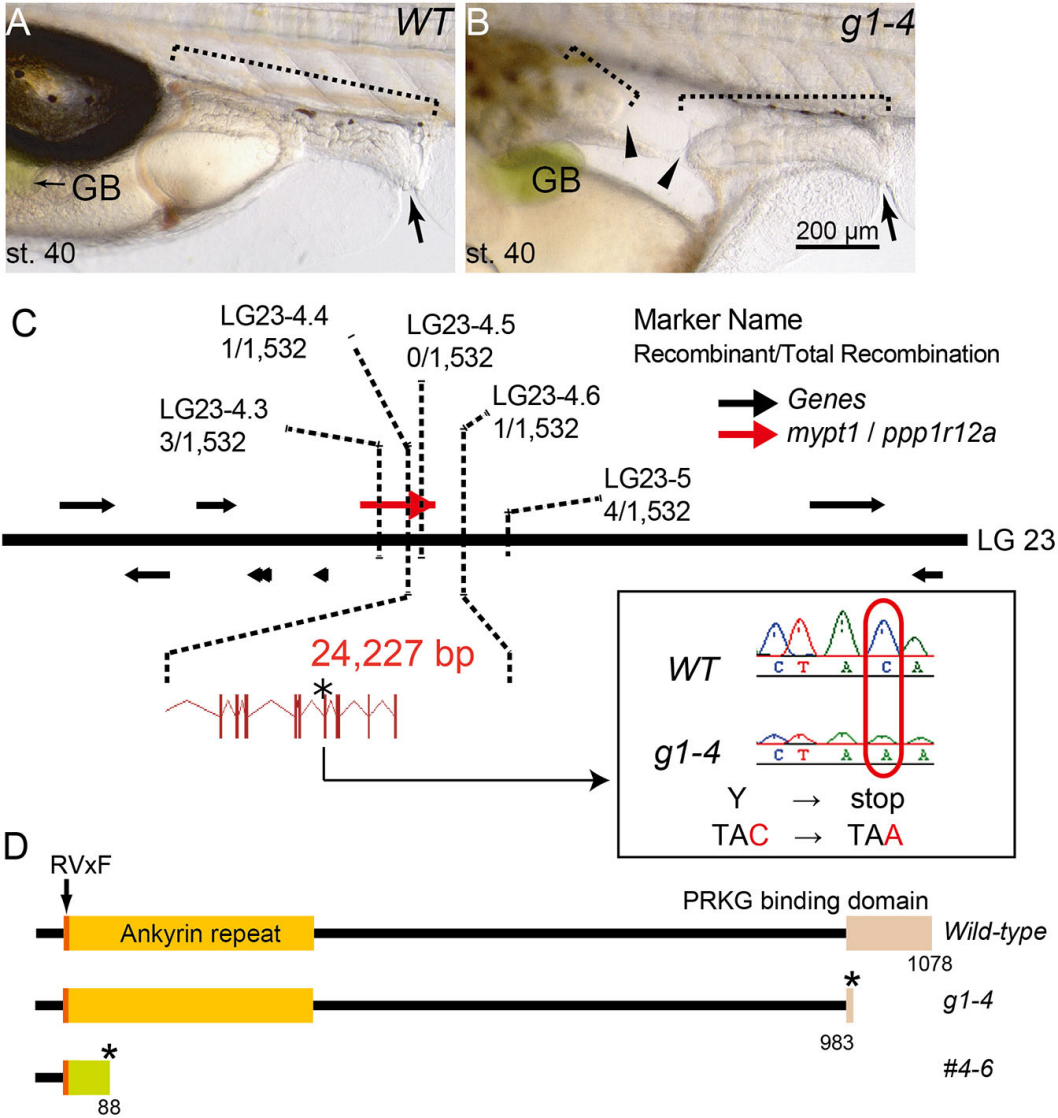
**Characterisation of medaka intestinal atresia (IA) mutant, *g1-4*.** (A,B) Lateral views of wild-type (WT; A) and *mypt1* mutant (*g1-4*) (B) embryos at stage (st.) 40. Note the development of IA in the mutant, represented by the blind ends of the intestine (arrowheads). Anterior is to the left. Arrows indicate cloaca; dotted line brackets indicate intestine. GB, gallbladder. Scale bar: 200 µm. (C) Schematic presentation of part of linkage group (LG)23. The *g1-4* locus is confined to a 0.13 cM interval between the markers LG23-4.4 and LG23-4.6. Inset shows a chromatogram of a point mutation in *g1-4*, which gives rise to a premature stop codon. Black arrows indicate annotated genes; red arrow indicates the *mypt1/ppp1r12a* gene; asterisk indicates the position of the mutation resulting in a premature stop codon. (D) Schematics outlining Mypt1 domains of WT (top), *g1-4* (middle) and *mypt1^#4-6^* (bottom) proteins. Asterisks indicate premature translation termination. In *mypt1^#4-6^*, an 11-nucleotide deletion with a seven-nucleotide insertion cause a frame shift (green), leading to truncation.

The *g1-4* medaka mutant was isolated as a recessive lethal line through our ENU mutagenesis screening for mutations affecting embryonic development and organogenesis (Yokoi et al., 2007). Examination of homozygous mutant embryos revealed the presence of IA, which developed at st. 32–33, although the penetrance rate of the IA phenotype varied and did not reach 100% (Table 1). In adult medaka, small and large intestines were distinguishable by morphological characteristics and distinct gene expression profiles (Aghaallaei et al., 2016). However, we could not distinguish small from large intestine in the embryos either morphologically or by the expression of known molecular markers. Therefore, hereafter, we refer to the region between the liver bud and the cloaca simply as the intestine. Despite the existence of IA, the remaining intestinal tissue appeared to be properly developed at st. 40, the hatching stage (Fig. 1A,B).

**Table 1.** Variation in penetrance among *g1-4* pairs

Fish pair		IA	Total	%
Female 1	Male 1	1	37	2.7
Female 2	Male 2	8	45	17.8
Female 2	Male 3	8	36	22.2
Female 3	Male 2	5	30	16.7
Female 4	Male 3	2	20	10.0
Female 5	Male 1	4	45	8.9

‘IA’ column shows the number of embryos that develop intestinal atresia. ‘Total’ column shows the number of embryos after deduction of those for which intestinal phenotype could not be determined (dead or abnormal development).

### The *g1-4* allele encodes *mypt1*

We hypothesised that the inheritance mode of the *g1-4* mutant was recessive, and, to identify the affected locus, we performed positional cloning. Initial mapping using M-marker analysis placed the mutation on linkage group (LG)23 (Kimura et al., 2004). We then conducted high-resolution linkage analysis using an F2 mapping panel comprising 766 embryos. This analysis narrowed the *g1-4* locus to a 0.13 cM interval between the markers LG23-4.4 and LG23-4.6, corresponding to a genomic region of 24.227 kb (Fig. 1C). Within this region, only one gene, *mypt1*, also known as *protein phosphatase 1 regulatory subunit 12a* (*ppp1r12a*) was identified in the medaka reference genome.

The open reading frame of medaka *mypt1* (3234 bp; LC662536) consists of 25 exons and encodes a predicted protein of 1078 amino acids that is closely related to human MYPT1 (NP_001137357.1). Medaka Mypt1 has three highly conserved domains – an RVxF motif, an ankyrin repeat and a protein kinase cGMP-dependent (Prkg) interacting domain (Fig. 1D). Sequencing the *g1-4* allele revealed a C-to-A transversion in exon 22 (C2952A), resulting in a premature stop codon (Fig. 1C,D). The predicted premature termination occurs within the Prkg-interacting domain and eliminates the C-terminal leucine-zipper (LZ) motif, which is essential for interaction with Prkg1α (Grassie et al., 2011; Surks et al., 1999). As interaction with Prkg1α is essential for actomyosin activation, the C2952A mutation is expected to impair the function of Mypt1.

To confirm whether the *mypt1* mutation causes the IA phenotype observed in the *g1-4* mutants, we tried to rescue the defect using wild-type (WT) *mypt1* mRNA. However, for unknown reasons, we could not synthesise the full-length *mypt1* mRNA *in vitro*. Alternatively, we generated *mypt1* knockout medaka using the CRISPR/Cas9 system (Ansai and Kinoshita, 2014). We isolated a mutant line possessing an 11-nucleotide deletion with a seven-nucleotide insertion (*mypt1^#4-6^*, c.127_137delinsTCTGTAT; Fig. 1D; Fig. S1A,B). This mutation creates a transcriptional frame shift that alters 45 codons before introducing a premature stop codon at the 88th codon. Crosses between F1 *mypt1^#4-6^* heterozygotes yielded embryos displaying IA at a frequency of 23.7% (*n*=59), and we successfully established a stable mutant line. In the F2 generation, the penetrance of the IA phenotype varied among siblings, similar to the original *g1-4* mutant line (Table 2), with ∼15–30% of total siblings exhibiting IA – higher penetrance than in the original mutant. For further phenotypic analysis, we used this newly generated mutant line, *mypt1^#4-6^*. We could also see dilation of the intestine upstream of the atretic region at the hutching stage, a hallmark commonly associated with IA in *mypt1^#4-6^* embryos (Fig. S2A–D). Taken together, we concluded that *mypt1* mutation is responsible for the IA phenotype.

**Table 2.** Variation in penetrance among *mypt1^#4-6^* pairs

Generation	Fish pair		IA	Total	%
	Female	Male			
F1	16	17	14	59	23.7
F2	33	34	19	97	19.6
	35	36	9	29	31.0
	37	38	8	30	26.7
	39	40	13	71	18.3
	41	43	13	84	15.5
	44	45	9	44	20.5
	46	47	17	62	27.4
	48	49	13	90	14.4
	50	51	12	72	16.7
	54	55	5	39	12.8
	56	57	15	83	18.1
F2 average			133	701	19.0

‘IA’ column shows the number of embryos that develop intestinal atresia. ‘Total’ column shows the number of embryos after deduction of those for which intestinal phenotype could not be determined (dead or abnormal development).

### Intestinal development is largely preserved in *mypt1^#4-6^* mutants, except at the atresia lesion

To investigate the phenotype of the *mypt1^#4-6^* mutant, we performed histological analyses on st. 40 larvae, immediately after hatching. Serial cross-sections were prepared from WT (*n*=3) and *mypt1^#4-6^* mutant larvae (*n*=3) and compared. The mutants exhibited IA, whereas other organs appeared to develop normally (Fig. S2E–P). To further assess the epithelial architecture at earlier stages, we performed conventional histological analysis using plastic sections at st. 32. These sections showed that the embryonic intestine appeared morphologically intact in *mypt1^#4-6^* mutants, except at the atretic lesion. The mesenchymal cells, which give rise to SM and connective tissue, surrounded the endodermal epithelium similarly to WT, and this organisation was also preserved in unaffected regions of the mutant intestine (Fig. S2Q–X) (Wallace et al., 2005a). Homozygous mutants were generally lethal, with a few individuals surviving to adulthood. Most homozygous mutants died after hatching. Thus, IA is very likely one of the major causes of lethality, as affected larvae are unable to feed after hatching. However, we cannot rule out the possibility that other factors contribute to lethality. Importantly, although most survivors exhibited poor health, some were indistinguishable from WT fish (Movies 1 and 2).

**Fig. 2.**
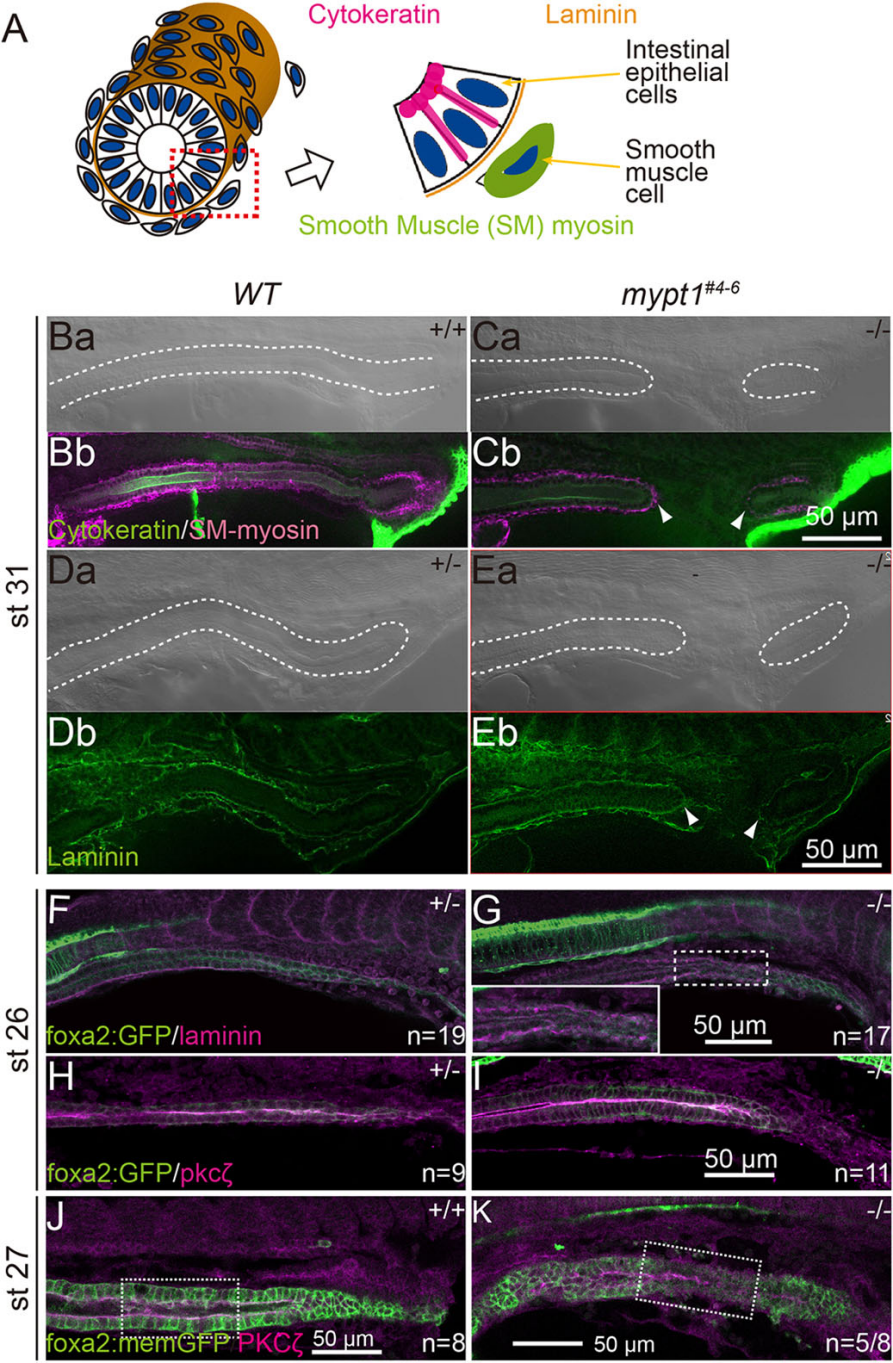
**Apico-basolateral polarity markers are maintained in *mypt1* mutants.** (A) Schematic diagram of the distribution of cytokeratin, laminin and smooth muscle (SM) myosin in the developing intestine. (Ba–K) Whole-mount immunofluorescence micrographs of cytokeratin and SM-myosin (Ba–Cb), laminin (Da–G) and Pkcζ (H-K) in WT (Ba,Bb,Da,Db,F,H,J) and *mypt1^#4-6^* (Ca,Cb,Ea,Eb,G,I,K) as left lateral view, optical sections. White dashed lines mark the outline of the intestine in a differential interference contrast micrograph corresponding to a fluorescence image. Note that the basement membrane was fragmented in the mutant embryos at st. 26 before IA onset (F, *n*=19; G, *n*=17/18). (H–K) Immunohistochemistry for Pkcζ in st. 26 (H,I) and st. 27 (J,K) embryo intestines. The epithelium of the intestine was visualised with *Tg*[*foxa2:memGFP*] (H, *n*=9; I, *n*=10; J, *n*=8; K, *n*=8). The apical localisation of Pkcζ was not disturbed in *mypt1* mutants at st. 26 (I). A partial loss of apical Pkcζ localisation was observed at st. 27 (K, *n*=5/8). See magnified images in Fig. S6. Arrowheads indicate blind ends of IA. Scale bars: 50 µm.

Cytokeratin, a marker of epithelial cells and intermediate filament protein, predominantly localises to the apical plasma membrane in polarised epithelia (Fig. 2A) (Yang et al., 2020). Consistent with this, the intestinal epithelium of st. 31 WT embryos showed strong apical localisation of cytokeratin (Fig. 2Bb). In *mypt1^#4-6^* mutants, the intestinal epithelium – excluding the atretic lesion – displayed cytokeratin distribution comparable to that of WT (Fig. 2Cb).

SM cells, labelled by SM-specific myosin (SM-myosin), properly surrounded the intestinal epithelium in both *WT* and *mypt1^#4-6^* embryos at st. 31 (Fig. 2Ba–Cb). Notably, the basement membrane (BM), labelled by laminin, was present beneath the epithelium even at the blind-end of the atresia in the mutant (Fig. 2Da–Eb). These findings indicate that the fundamental structure of the gut – including epithelial apico-basolateral polarity, SM layer and BM – is preserved normally in the region outside the atresia. This is consistent with observations in human IA cases.

**Fig. 3.**
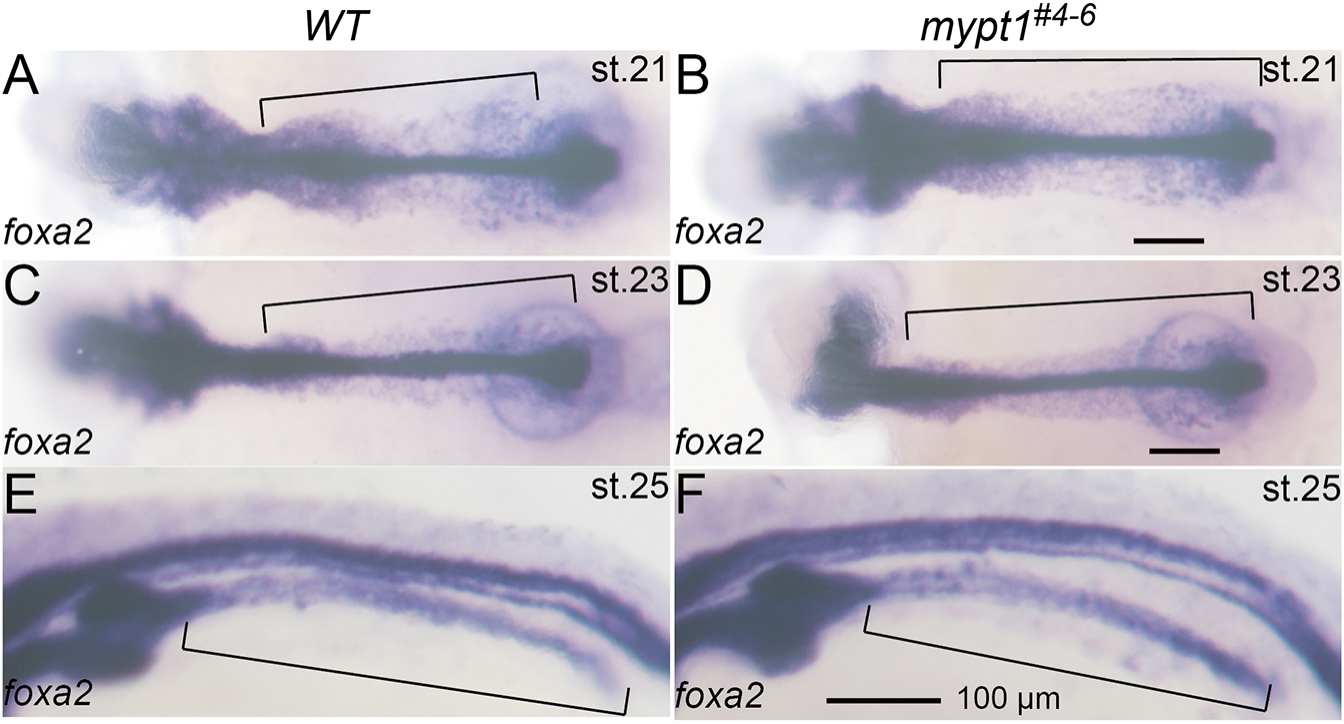
**Endodermal migration to form the intestinal anlage is intact in *mypt1* mutants.** (A–F) WT (A,C,E, *n*=10) and *mypt1* mutant (B,D,F, *n*=10) embryos were subjected to whole-mount *in situ* hybridisation (WISH) for *foxa2*. Dorsal (A–D) and dorsolateral views (E,F) are shown. Embryonic stages are indicated in the top right. Brackets mark endoderm and intestinal anlage. Scale bars: 100 µm.

To elucidate the sequence of events that lead to atresia formation in *mypt1^#4-6^* embryos, we carefully monitored live embryos but could not detect morphological signs of IA until st. 28. We first examined endodermal development, as the endoderm gives rise to the intestine, to determine whether endodermal defects were present before or during intestinal tube formation. Expression of *foxa2*, a marker of the endoderm and its derivatives including the intestine (Kobayashi et al., 2006), was indistinguishable between WT and *mypt1^#4-6^* embryos up to st. 25 (Fig. 3), suggesting that early endoderm formation and migration are not disrupted in the mutant.

Importantly, at st. 26 – when intestinal tube formation is completed (Kobayashi et al., 2006) – fragmentation of BM was observed in 17 of 18 *mypt1^#4-6^* mutants (Fig. 2G), whereas none of the 19 WT embryos showed such fragmentation (Fig. 2F; Fig. S3). These results indicate that molecular events leading to IA had already begun by st. 26, even though no obvious morphological anomalies were yet visible.

**Fig. 4.**
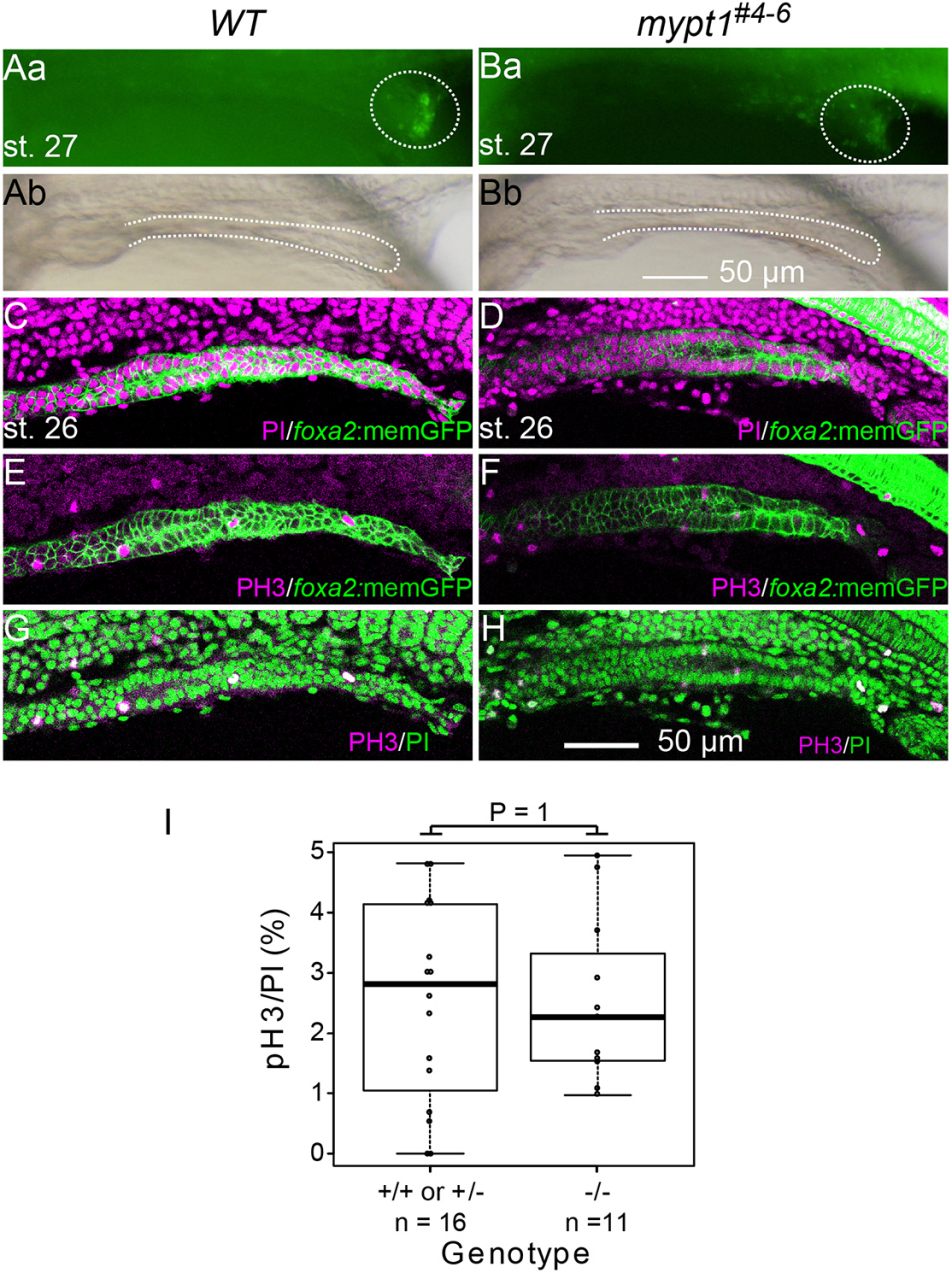
**Cell death and cell proliferation are not involved in IA development.** (Aa–H) Cell death and cell proliferation in the intestine are not significantly altered in *mypt1* mutants. (Aa,Ba) Acridine Orange staining of WT embryos (Aa, *n*=12) and *mypt1* mutants (Ba, *n*=5) at st. 27. (Ab,Bb) Brightfield images corresponding to Aa and Ba, respectively. Dotted ellipses mark apoptotic cells surrounding the cloacal opening, where apoptosis is normally observed (Parkin et al., 2009). (C–H) WT (C,E,G) and *mypt1* mutant (D,F,H) embryos at st. 26 were stained with anti-phosphorylated histone H3 (PH3) antibody and/or propidium iodide (PI). Scale bars: 50 µm. (I) Quantification of PH3-positive cell ratio to total cell number in the intestinal region was not significantly different between WT (*n*=16) and *mypt1* mutants (*n*=11; chi-square test, *P*=1). Boxes represent the 25th–75th percentiles, and the median is indicated. The whiskers show the minimum and maximum values.

To investigate whether epithelial disintegration due to apoptosis or altered proliferation contributed to BM fragmentation, we performed Acridine Orange (AO) staining from st. 26 to st. 28. This revealed no detectable apoptotic cells in either WT or *mypt1^#4-6^* intestines (Fig. 4Aa–Bb; Fig. S4). Terminal deoxynucleotidyl transferase-mediated dUTP nick-end labelling (TUNEL) assays also showed no significant difference between *WT* and mutant embryos (Fig. S5). Furthermore, proliferation, assessed by anti-phosphorylated histone H3 (PH3) staining, showed no significant difference in the number of mitotic cells between WT and mutant intestines (Fig. 4E–I). These data suggest that neither apoptosis nor altered proliferation underlies the BM fragmentation and IA in the mutant.

**Fig. 5.**
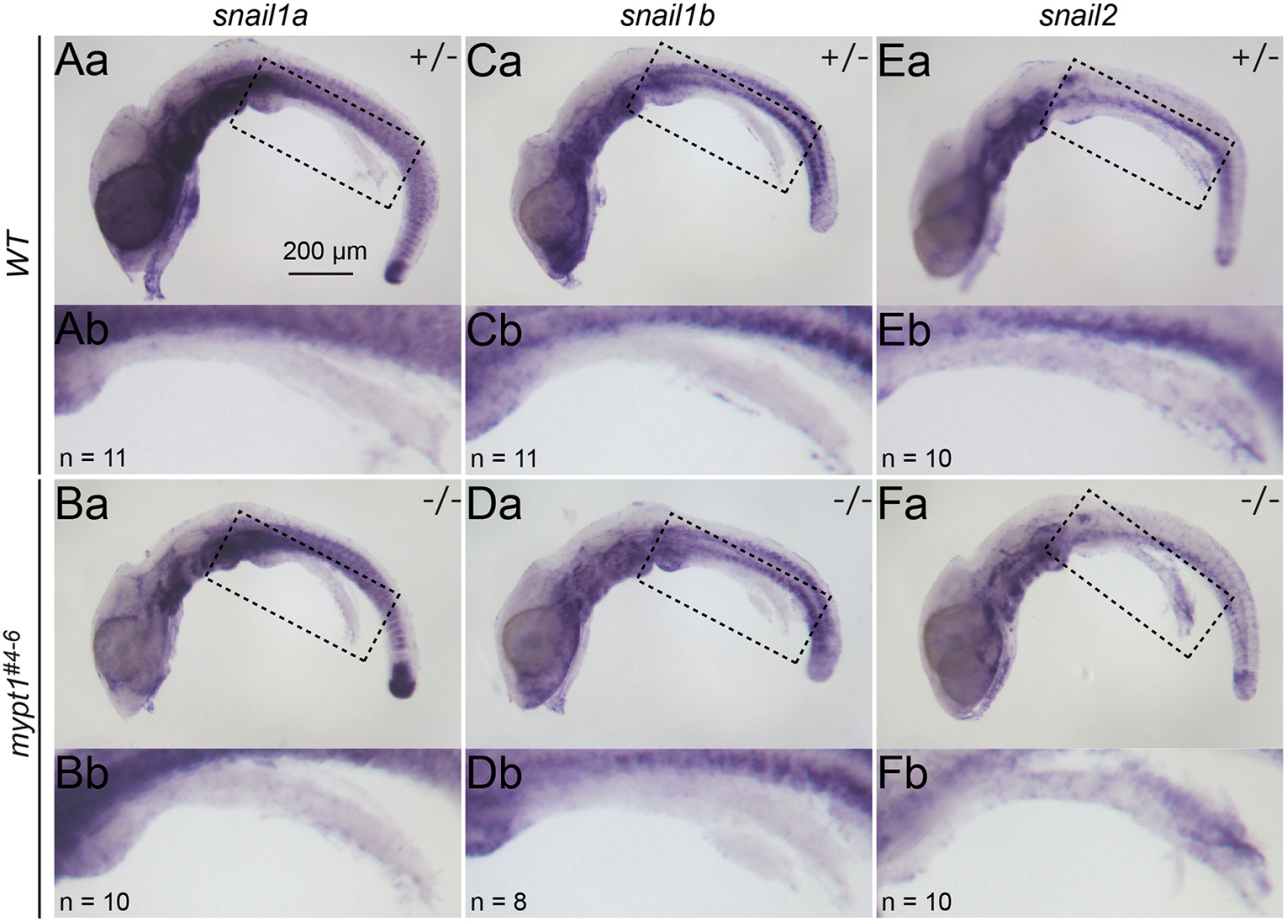
**Epithelial–mesenchymal transition (EMT) is not involved in IA formation in *mypt1* mutants.** (Aa–Fb) Lateral views of st. 26 embryos subjected to WISH to detect EMT markers in WT (Aa,Ab,Ca,Cb,Ea,Eb) and *mypt1* mutants (Ba,Bb,Da,Db,Fa,Fb): *snai1a* (Aa–Bb), *snai1b* (Ca–Db) and *snai2* (Ea–Fb). To enhance visibility, the intestine was detached from the dorsal mesentery. Dashed line boxes in Aa–Fa are enlarged in Ab–Fb, respectively. The number of samples examined is indicated in the bottom-left corner. Scale bar: 200 µm.

Although apico-basolateral polarity was preserved in the gut epithelium at st. 26, localised epithelial–mesenchymal transition (EMT) could hypothetically result in loss of polarity and BM fragmentation, as occurs during gastrulation (Savagner, 2015). To explore this possibility, we examined the expression of EMT-inducing transcription factors – *snai1a*, *snai1b* and *snai2* (Scheibner et al., 2021) – via whole-mount *in situ* hybridisation (WISH). None of these genes were expressed in the intestine of either WT or mutant embryos at st. 26 (Fig. 5Aa–Fb). Consistently, the subcellular localisation of Pkcζ, an apical epithelial marker, was not disrupted in *mypt1^#4-6^* intestines at this stage (Fig. 2H,I). At st. 27, however, a subset of mutant embryos exhibited a partial loss of apical Pkcζ localisation (Fig. 2K; Fig. S6; *n*=5/8). Notably, epithelial architecture was already perturbed at this stage, as indicated by disturbed distribution of membrane-bound GFP signals, making it difficult to determine whether the loss of epithelial polarity represents a primary defect or a secondary consequence of IA development. At st. 28, further deterioration of epithelial integrity was evident: in WT embryos, E-cadherin was localised to cell–cell junctions (Fig. S7A), whereas in *mypt1^#4-6^* mutants, E-cadherin signals colocalised with membrane-bound GFP in constricted epithelial regions in some embryos (Fig. S7B), but were markedly reduced or absent in others (Fig. S7C). Together, these observations indicate that BM fragmentation and IA formation in *mypt1^#4-6^* embryos are not driven by EMT, but are instead associated with a progressive and heterogeneous loss of epithelial polarity and junctional integrity during later stages of intestinal morphogenesis.

### Mypt family members are unlikely to compensate for the loss of Mypt1 in a tissue-specific manner

In zebrafish, *mypt1* mutations cause a spectrum of morphogenetic abnormalities, including defects in liver and pancreas development, dilation of brain ventricles and mispositioning of motoneurons (Bremer and Granato, 2016; Dong et al., 2019; Gutzman and Sive, 2010; Huang et al., 2008). In contrast, these phenotypes were not observed in *mypt1* mutant medaka, suggesting possible species-specific differences in gene function or redundancy within the Mypt gene family. To explore whether this phenotypic divergence could be explained by differential expression or functional compensation by other Mypt family members, we identified Mypt homologues in the medaka genome and analysed their spatial expression patterns by WISH.

In humans, the myosin phosphatase regulatory subunit family consists of *MYPT1* (*PPP1R12A*), *MYPT2* (*PPP1R12B*) and *MBS85* (*PPP1R12C*) (Gutzman and Sive, 2010; Huang et al., 2008). To determine whether other Mypt family genes might compensate for the loss of *mypt1* function in tissues other than the intestine in medaka mutants, we searched the medaka genome and identified three members of the Mypt family: *mypt1*, *mbs85*, and a novel gene that diverges from the ancestral lineage shared by *mypt1* and *mypt2*, which we designated *mypt1/2-related* (*mypt1/2-r*) (Table 3; Fig. S8). Notably, no clear *mypt2* orthologue was found in the medaka genome. Although medaka possesses a gene annotated as *mypt3*, this gene belongs to the Ppp1r16 family rather than the Ppp1r12 family and was therefore excluded from further analysis.

**Table 3.** List of protein sequences used for CLUSTALW analysis

Species	Protein symbol	Accession number
*Homo sapiens*	MYPT1	NP_002471.1
*Gallus gallus*	Mypt1	NP_990454.1
*Danio rerio*	Mypt1	NP_001003870.2
*Oryzias latipes*	Mypt1	XP_023807904.1
*Homo sapiens*	MYPT2	NP_002472.2
*Gallus gallus*	Mypt2	XP_040508700.1
*Homo sapiens*	MBS85	NP_060077.1
*Gallus gallus*	Mbs85	XP_025002065.1
*Danio rerio*	Mbs85	NP_001071047.1
*Oryzias latipes*	Mbs85	XP_023810293.1
*Danio rerio*	Mypt1/2-r	XP_002666820.2
*Oryzias latipes*	Mypt1/2-r	XP_020559658.2
*Homo sapiens*	MYPT3	NP_001316373.1
*Danio rerio*	Mypt3	XP_001334473.1
*Oryzias latipes*	Mypt3	XP_011487151.1
*Homo sapiens*	TIMAP	NP_056383.1
*Gallus gallus*	Timap	NP_001026022.2
*Danio rerio*	Timap	XP_001340092.6
*Oryzias latipes*	Timap	XP_004068819.1
*Drosophila melanogaster*	Mbs	NP_001261919.1
*Caenorhabditis elegans*	MEL-11	AAB47273.1

**Fig. 6.**
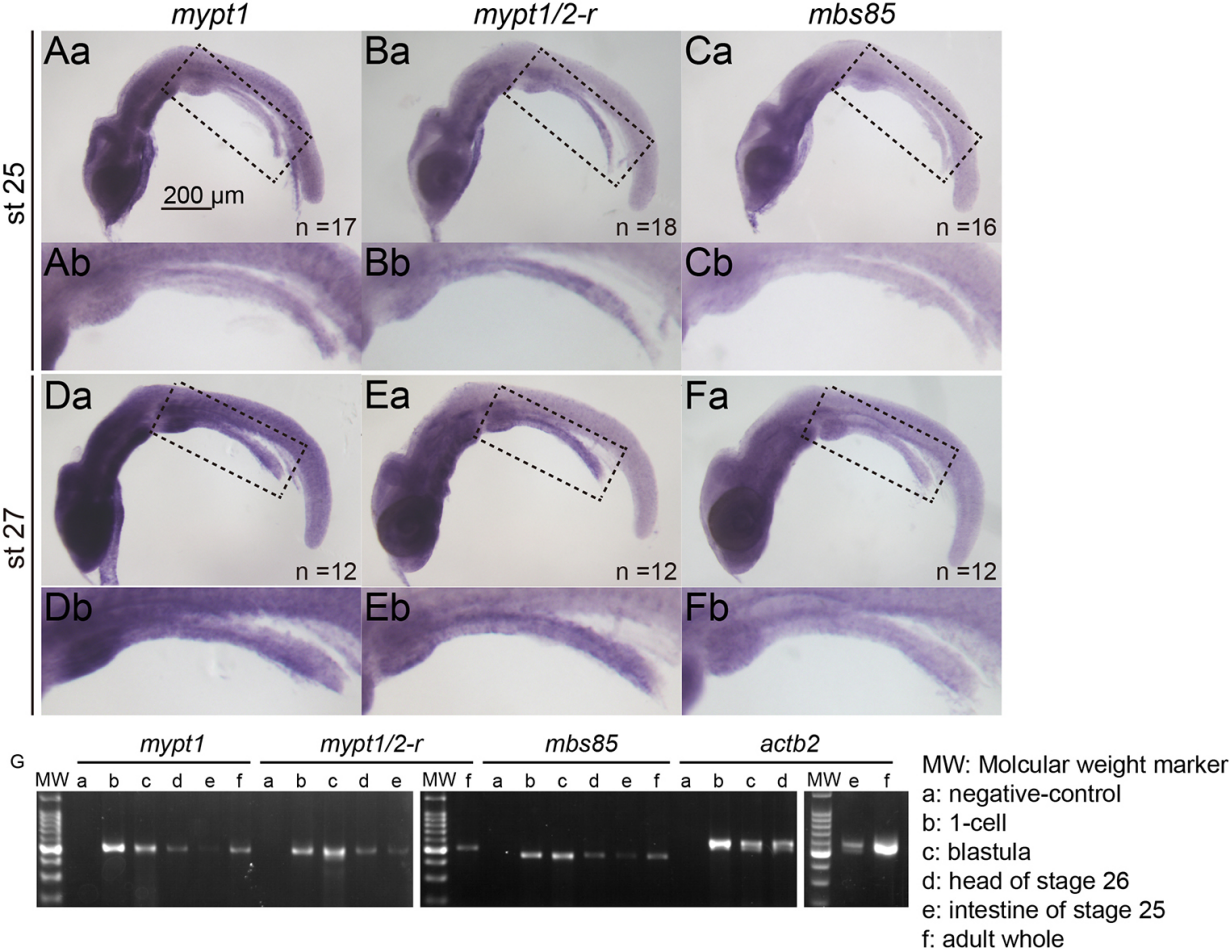
**Expression patterns of Mypt family genes.** (Aa–Fb) WISH of *mypt1* (Aa,Ab,Da,Db), *mypt1/2-r* (Ba,Bb,Ea,Eb) and *mbs85* (Ca,Cb,Fa,Fb) at st. 25 (Aa–Cb) and st. 27 (Da–Fb) in WT. Dashed line boxes in Aa–Fa are enlarged in Ab–Fb, respectively. The number of samples examined is indicated in the bottom-right corner. Scale bar: 200 µm. (G) RT-PCR analysis. PCRs using first-strand complementary DNA from (a) negative control, (b) one-cell stage, (c) blastula stage, (d) head region at st. 26, (e) intestine region at st. 25 and (f) adult whole body. MW, molecular mass marker (DM2100, SMOBIO).

We examined the expression patterns of *mypt1*, *mypt1/2-r* and *mbs85* by WISH. Both *mypt1* and *mypt1/2-r* were ubiquitously expressed at low levels, with slightly elevated expression in the head region. *mbs85* exhibited lower overall expression than the other two genes but displayed a similar spatial pattern. No significant differential expression was detected in the developing intestine (Fig. 6Aa–Fb). Reverse transcription PCR (RT-PCR) analysis of dissected head and intestinal tissues confirmed the expression of all three genes, supporting their broad, low-level distribution. These findings suggest that the intestinal specificity of the atresia phenotype in *mypt1* mutants is not due to tissue-specific expression of Mypt family genes.

### Mypt1 is maternally expressed

Previous studies in zebrafish have shown that maternal knockdown of *mypt1* using translation-blocking morpholinos leads to convergent extension (CE) defects during gastrulation (Weiser et al., 2009). In contrast, such defects were not observed in the *mypt1^#4-6^* mutant medaka line. To investigate whether maternal *mypt1* transcripts contribute to early embryogenesis and mask early developmental phenotypes, we performed RT-PCR on one-cell-stage embryos. The results confirmed the presence of *mypt1* transcripts at this stage, indicating that maternal *mypt1* mRNA is supplied during oogenesis. This maternal expression is likely to compensate for the absence of zygotic *mypt1*, thereby preventing CE defects in *mypt1^#4-6^* mutants (Fig. 6G).

### Actomyosin regulation is perturbed in *mypt1* mutant intestine

Phosphorylation of Mrlc governs the contractility of NMII (Ito et al., 2004). Dephosphorylated Mrlc reduces NMII activity and cytoskeletal contractility, whereas pMrlc activates NMII. Thus, NMII-driven contractility is determined by the balance between phosphorylation mediated by myosin light chain kinase (Mlck) and dephosphorylation by MLCP. *mypt1* encodes the regulatory subunit of MLCP that is essential for its enzymatic function (Ito et al., 2004). Therefore, elevated levels of pMrlc are expected in *mypt1* mutants.

**Fig. 7.**
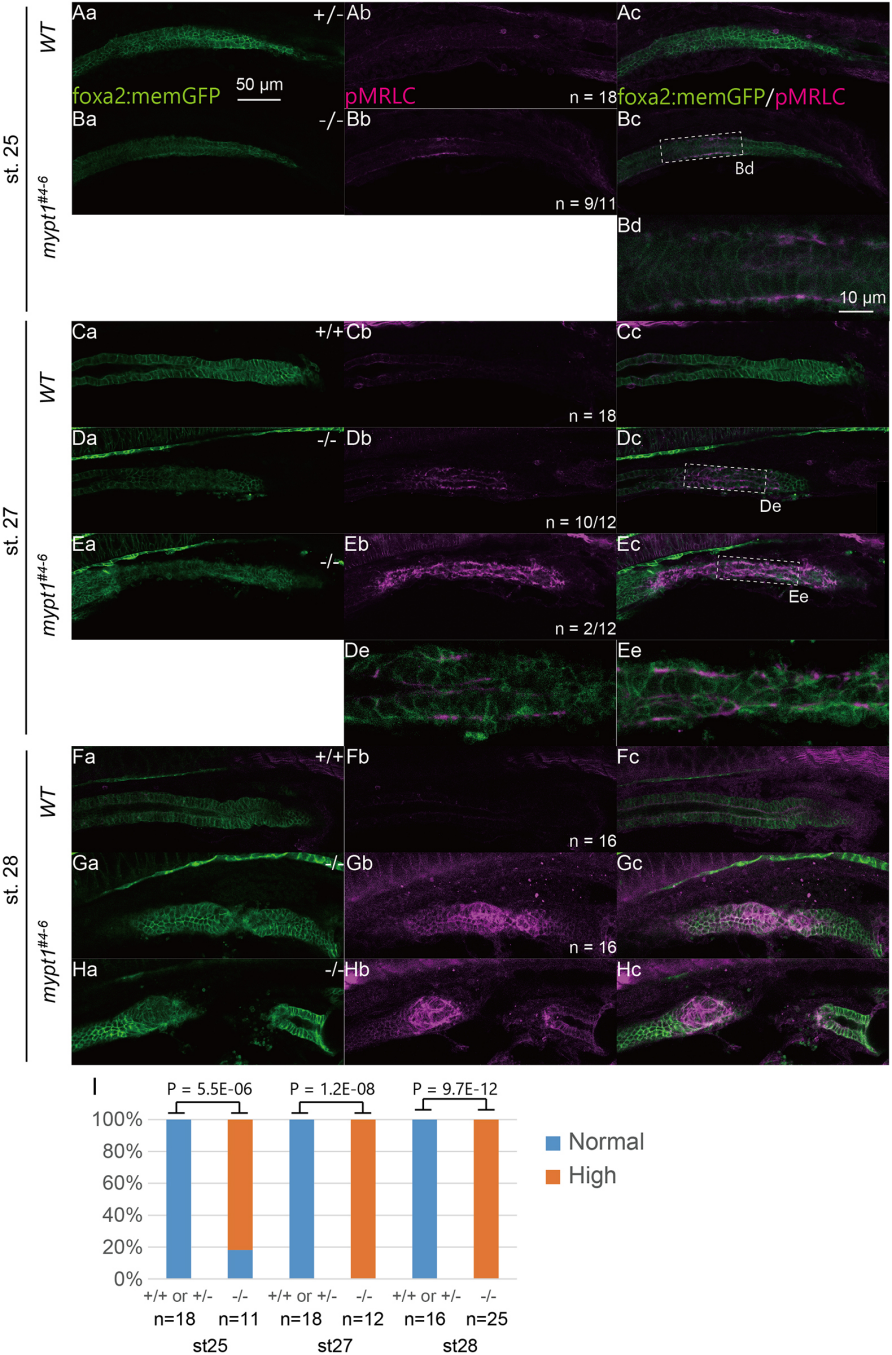
**pMrlc is abnormally activated in the developing intestine of *mypt1* mutants.** (A–H) Fluorescence micrographs of pMrlc for WT (Aa–Ac,Ca–Cc,Fa–Fc) and *mypt1^#4-6^* (Ba–Bc,Da–Dc,Ea–Ec,Ga–Gc,Ha–Hc). st. 25 embryos are shown in Aa–Bc, st. 27 embryos are shown in Ca–Ec, and st. 28 embryos are shown in Fa–Hc. Note significant accumulation of pMrlc in mutant intestinal epithelium (Bb,Db,Eb,Gb,Hb). Dashed line boxes in Bc, Dc and Ec are enlarged in Bd, Dd and Ed, respectively. Scale bars: 50 µm (10 µm in Bd,Dd,Ed). (I) The proportion of embryos possessing an abnormal increase in pMrlc in embryonic intestine (Fisher's exact test).

In WT embryos, pMrlc exhibited weak and diffuse localisation at both the apical and basal surfaces of intestinal epithelial cells (Fig. 7Aa–Ac, st. 25; Fig. 7Ca–Cc, st. 27; Fig. 7Fa–Fc, st. 28). As predicted, *mypt1* mutants displayed increased pMrlc levels at st. 25 compared with WT embryos (Fig. 7Ba–Bd), whereas no apparent difference was observed at st. 24 (Fig. S9). Notably, pMrlc upregulation was not uniform along the intestinal axis but was enriched in discrete regions corresponding to presumptive IA lesions (Fig. 7Ba–Bd). Higher-magnification analysis revealed that this increased pMrlc signal was localised specifically to the intestinal epithelium, as marked by *foxa2:memGFP* expression (Fig. 7Bd).

By st. 27, this abnormal expression pattern became more pronounced (Fig. 7Da–De), and, in a subset of embryos, strong pMrlc expression was detected along the entire intestinal tract (Fig. 7Ea–Ee; two of 12 samples). At st. 28, the progression of IA varied among embryos (Fig. 7Ga–Hc). In specimens in which IA was imminent, elevated pMrlc signals were detected around the constricted intestinal region (Fig. 7Ga–Gc), whereas similar enrichment was observed in embryos in which the intestinal epithelium appeared to have recently ruptured (Fig. 7Ha–Hc). Collectively, these findings indicate that pMrlc regulation is markedly altered in *mypt1* mutants from st. 25 onward, particularly in regions predisposed to IA formation (Fig. 7Ga–Gc).

Because pMrlc binds NMII and promotes actin–myosin interaction, increased and mislocalised pMrlc is likely to enhance actomyosin contractility and redistribute mechanical forces within the tissues (Vicente-Manzanares et al., 2009). In WT intestinal epithelial cells, F-actin was predominantly localised to the cortical region beneath the plasma membrane, with modest enrichment at the apical surface (Fig. S10A,C,E). In contrast, although overall intestinal lumen morphology appeared comparable between WT and *mypt1* mutants, F-actin accumulation was markedly elevated at both the apical and basal cortical regions in *mypt1* mutant epithelial cells (Fig. S10B,D,F,G). This phenotype is consistent with previous reports showing that increased pMrlc enhances actin filament assembly (Gutzman and Sive, 2010), whereas reduced pMrlc leads to decreased F-actin levels (Bavaria et al., 2011; Ray et al., 2007).

Together, these results indicate that loss of *mypt1* function leads to upregulation and mislocalisation of pMrlc, suggesting hyperactive actomyosin contractility of intestinal epithelium. This aberrant mechanical activity may compromise epithelial integrity and contribute to IA development in *mypt1* mutant embryos. Importantly, simultaneous staining for laminin and F-actin revealed that regions of BM fragmentation overlapped with areas of elevated F-actin signals (Fig. S11), suggesting a close association between actomyosin activation and BM disruption. These observations support a model in which aberrant mechanical forces generated by dysregulated actomyosin contractility compromise epithelial integrity, promote BM fragmentation and, ultimately, contribute to the development of intestinal atresia in *mypt1* mutant embryos. The cellular events preceding IA development are summarised in Table 4.

**Table 4.** Summary of molecular abnormalities associated with intestinal atresia

Stage	IA	pMrlc	F-actin	Laminin	Pkcζ
24	−	−	−	nd	nd
25	−	9/11	7/12	nd	nd
26	−	8/8	nd	17/18	0/11
27	−	12/12	7/7	nd	5/8
28	±	14/14	14/14	nd	nd
	+	11/11	11/11		

IA, intestinal atresia; nd, not determined. ‘pMrlc’ and ‘F-actin’ columns show the number of embryos showing abnormally elevated pMrlc/F-actin signals/total number of embryos observed. ‘Laminin’ column shows the number of embryos exhibiting fragmented laminin signals/total number of embryos observed. ‘Pkcζ’ column shows the number of embryos showing loss of apical Pkcζ localisation/total number of embryos observed.

### SM-myosin function partially contributes to the development of IA

Given that Mypt1 regulates SM-myosin activity, abnormalities in the SM layer surrounding the intestinal epithelium could potentially contribute to the pathogenesis of IA. In the medaka genome, we identified two homologues of *MYH11*: *myh11a* on LG1 and *myh11b* on LG8. However, RT-PCR analysis at 2 days post-fertilisation (dpf) revealed expression of *myh11a* only (Fig. S12), prompting us to focus our investigation on *myh11a*.

To disrupt *myh11a* function, we designed three overlapping single-guide RNAs (sgRNAs) targeting the ATP-binding domain of *myh11a* (Fig. S13A). These sgRNAs were injected into medaka embryos at the one- to two-cell stage, either alone or in combination with Cas9 nuclease. To confirm the effect of CRISPR-mediated knockout, we performed immunohistochemical analysis of SM-myosin expression at a developmental stage when the SM layer is well established. In the embryos injected without Cas9 at 6 dpf, SM-myosin was robustly detected in the SM layer surrounding the intestinal epithelium (Fig. S13B; six of six embryos). In contrast, embryos co-injected with Cas9 exhibited either a complete loss of SM-myosin expression (Fig. S13C; two of six embryos) or an aberrant, patchy expression pattern (Fig. S13D; four of six embryos), indicating effective disruption of functional SM-myosin.

We then assessed whether IA still occurred under these SM-myosin-deficient conditions in *mypt1^#4-6^* homozygous mutant embryos. Notably, IA was absent in four of ten *mypt1^#4-6^* embryos co-injected with *myh11a* sgRNAs and Cas9, whereas all nine embryos injected with *myh11a* sgRNAs alone (without Cas9) developed IA (Table 5; Fig. S13E–G). To further validate the role of *myh11a*, we performed morpholino-mediated knockdown using a splice-blocking morpholino targeting the exon 2–intron 2 junction (Mo-*myh11a*-E2I2; Fig. S13H). RT-PCR confirmed efficient disruption of normal splicing (Fig. S13I), and partial rescue of IA was observed in morpholino-treated embryos (Table 6). These findings suggest that *myh11a*-dependent SM function contributes to IA pathogenesis.

**Table 5.** Intestinal atresia phenotype in homozygous *mypt1* mutant embryos injected with *myh11a* sgRNAs with or without Cas9 nuclease

Cas9(+)			Cas9(−)		
IA(+)	IA(−)	Total	IA(+)	IA(−)	Total
6	4	10	9	0	9

Embryos were injected at the one-cell stage with a mixture of three sgRNAs targeting *myh11a*, either alone or in combination with Cas9 protein. The presence or absence of intestinal atresia (IA) was scored in *mypt1* mutant homozygous embryos. Injection of sgRNAs with Cas9 partially rescued the IA phenotype, whereas sgRNAs injection alone had no significant effect.

**Table 6.** Intestinal atresia phenotype in homozygous *mypt1* mutant embryos injected with Mo-*myh11a*-E2I2 morpholino

Morpholino	Concentration (µM)	IA(+)	IA(−)	JNP	Total
Control	200	22	1	1	23
Mo-*myh11a*-E212	150	14	6	0	20
	200	21	9	11	41

JNP, judgement not possible. Embryos were injected at the one-cell stage with Mo-*myh11a*-E2I2 or control morpholinos. The presence or absence of IA was scored in *mypt1* mutant homozygous embryos. Injection of Mo-*myh11a*-E2I2 partially rescued the IA phenotype, whereas injection of control morpholino had no significant effect.

Importantly, at st. 28, when IA first becomes morphologically detectable, no obvious SM layer exhibiting Myh11a immunoreactivity was observed surrounding the intestinal epithelium (Fig. S14). This observation suggests that SM contraction is unlikely to be directly responsible for the initiation of IA. Instead, these findings suggest that *myh11a* exerts its effect through non-SM cell populations, potentially including intestinal epithelial cells, or via indirect, non-cell-autonomous mechanisms acting at earlier developmental stages. Taken together, *myh11a*-dependent SM function is unlikely to be the primary driver of IA formation, although it may contribute partially to disease penetrance.

### Augmented actomyosin contraction is responsible for IA in *mypt1* mutants

**Fig. 8.**
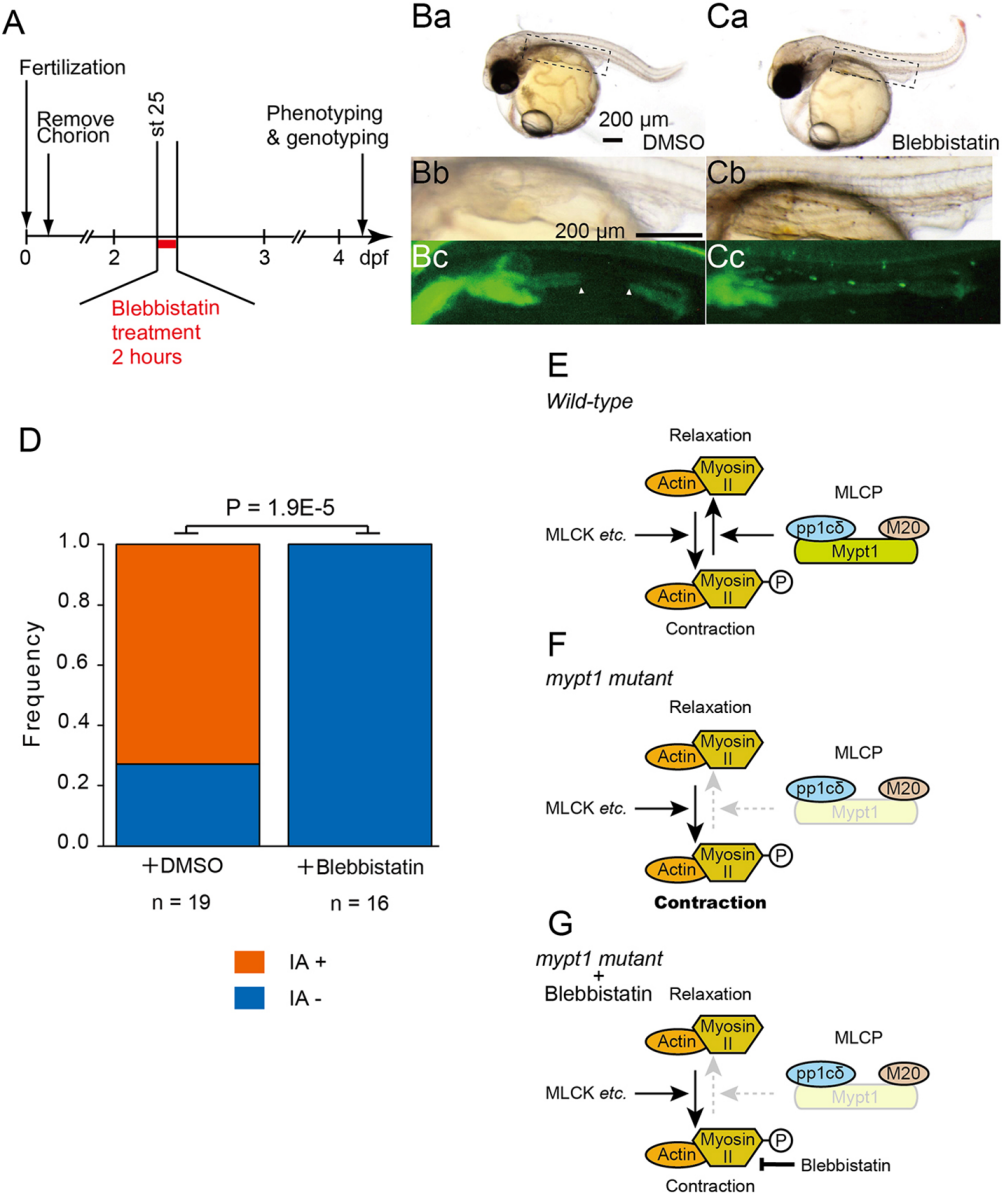
**Attenuation of actomyosin activation rescues IA phenotype in *mypt1* mutants.** (A) Experimental design for blebbistatin treatment. (Ba–Cb) Brightfield images of control (Ba,Bb) and blebbistatin-treated (Ca,Cb) *mypt1* mutants at 9 days post-fertilisation (dpf). Dashed line boxes in Ba and Ca are enlarged in Bb and Cb, respectively. Scale bars: 200 µm. (Bc,Cc) Corresponding fluorescent images of Bb and Cb, respectively. Arrowheads indicate IA. (D) Quantification of rescue of the IA phenotype in *mypt1^#4-6^* embryos following blebbistatin treatment (Fisher's exact test, *P*=1.9×10^–5^). (E) Diagram of non-muscle myosin II regulation. Mypt1 is a critical subunit of myosin light chain phosphatase (MLCP) and is essential for its function. Loss of Mypt1 leads to reduced MLCP activity, resulting in ectopic activation of actomyosin contraction. (F) Hypothetical molecular events following the loss of *mypt1* function. (G) Schematic representation of the molecular state under blebbistatin treatment. P, phosphorylation status of MRLC in non-muscle myosin II.

To further investigate whether enhanced cytoskeletal contractility contributes to the development of IA in *mypt1* mutants, we tested whether blebbistatin – an NMII inhibitor – could rescue the IA phenotype in *mypt1^#4-6^* mutant embryos. Embryos were treated with 50 µM blebbistatin at st. 25 for 2 h (Fig. 8A). Under these conditions, no major morphological abnormalities were observed other than a pronounced curly tail phenotype (Fig. 8Ba,Ca). Remarkably, IA was not observed in all treated *mypt1* mutant embryos (Fig. 8Cb–D), strongly suggesting that excessive actomyosin contractility is a key driver of IA formation.

## DISCUSSION

Here, we report a novel animal model of IA. Through ENU mutagenesis of medaka, we isolated the *g1-4* mutant, in which early intestinal development proceeds normally, but loss of epithelial integrity leads to IA after the intestinal lumen has formed. Positional cloning identified a loss-of-function mutation in the *mypt1* gene. A genome-edited *mypt1* mutant allele (*mypt1^#4-6^*) recapitulated the *g1-4* phenotype, confirming that loss of Mypt1 function is responsible for IA. In *mypt1* mutants, we observed elevated levels of pMrlc and abnormal accumulation of F-actin in the developing intestine (Fig. 7; Fig. S10). Treatment with blebbistatin, an NMII inhibitor, significantly suppressed IA in *mypt1* mutants (Fig. 8), suggesting that hyperactive cytoskeletal contraction is a likely cause of epithelial disruption and IA formation. Given the limited understanding of the molecular pathophysiology of human IA and the scarcity of appropriate animal models, the medaka *mypt1* mutant provides a valuable platform for elucidating the mechanisms underlying this congenital condition.

A long-standing hypothesis proposes that IA arises from an accidental vascular injury during development (Louw, 1959; Louw and Barnard, 1955). However, in our medaka model, no overt abnormalities in blood circulation were observed in *mypt1^#4-6^* embryos. Furthermore, previous studies have shown that blood cells rarely traverse through this vessel at st. 28 in WT embryos and that robust blood circulation in the digestive tract is not established until st. 30 (Fujita et al., 2006), which is after the onset of IA formation, indicating that vascular perfusion at this site is minimal at the relevant stage. Together, these observations argue against a vascular origin of IA in *mypt1* mutants and instead support non-vascular mechanisms, such as dysregulated actomyosin contractility and impaired epithelial integrity during intestinal morphogenesis.

The developmental role of Mypt1 has been documented in some species. In *Drosophila*, *mypt1* mutants have various developmental defects, including failure of dorsal closure (Mizuno et al., 2002; Tan et al., 2003). In these mutants, ectodermal cell sheet movement is impaired, and the subcellular localisation of actomyosin regulatory proteins, such as pMrlc and actin, is perturbed (Mizuno et al., 2002). In zebrafish, *mypt1* mutants display impaired migration of bone morphogenetic protein 2a (*bmp2a*)-expressing lateral plate mesoderm (LPM) cells, causing agenesis/hypoplasia of the liver and exocrine pancreas (Dong et al., 2019; Huang et al., 2008). Brain ventricle formation is also affected in the zebrafish mutant because of ectopically induced tension in the neuroepithelium layer (Gutzman and Sive, 2010). In these mutants, increased pMrlc levels and aberrant localisation of NMII and F-actin are observed in the neuroepithelium. Importantly, the upregulation and mislocalisation of actomyosin regulatory components – such as pMrlc, NMII and F-actin – are likely to be common features in *mypt1* mutants across species, including medaka. Furthermore, in the context of brain ventricle development in zebrafish, blebbistatin treatment to inhibit NMII successfully rescued the *mypt1* mutant phenotype. This supports the notion that ‘epithelial relaxation’ regulated by Mypt1 is critical for proper tube inflation, as occurs in brain ventricle formation. Our findings are consistent with the view that loss of *mypt1* function affects actomyosin dynamics during embryogenesis.

During embryonic development, BM is not a static scaffold but undergoes dynamic remodelling to accommodate and support tissue morphogenesis (Khalilgharibi and Mao, 2021). In multiple developing epithelial systems, BM exhibits local thinning, pore formation and spatial reorganisation, which are tightly coupled to changes in epithelial tissue geometry. For example, in a salivary gland branching model, Harunaga et al. (2014) demonstrated that BM perforation and translocation occur at sites of epithelial expansion and depend on myosin II-mediated contractility, as these BM dynamics were suppressed by blebbistatin treatment. Similarly, recent *in vivo* imaging studies in other epithelial tissues have shown that BM turnover and local reorganisation accompany epithelial shape changes during morphogenesis, highlighting the dynamic and adaptive nature of the BM (Wuergezhen et al., 2025). In our *mypt1* mutant embryos, BM fragmentation is observed prior to the overt morphological manifestation of IA and is accompanied by abnormal accumulation of F-actin and pMrlc within the intestinal epithelium. Given that blebbistatin treatment disrupts BM remodelling in both systems, these findings suggest that deregulated actomyosin activity destabilises the BM, leading to aberrant BM remodelling before the onset of tissue obstruction. Importantly, BM fragmentation in the *mypt1* mutant intestine is, therefore, unlikely to represent a secondary consequence of epithelial breakdown. Rather, it is consistent with a primary defect in actomyosin-dependent BM remodelling, analogous to dynamic BM behaviours reported in other developing epithelial tissues. Although the precise molecular mechanisms linking epithelial shape changes to BM remodelling remain an open question, our data indicate that compromised BM integrity precedes IA formation and may contribute to intestinal fragility, thereby predisposing the tissue to luminal obstruction.

Despite these commonalities, notable differences exist between *mypt1* mutants in medaka and previously reported *mypt1* mutants in zebrafish. IA was not observed in zebrafish mutants, whereas neither liver nor brain ventricle abnormalities were found in medaka mutants (Gutzman and Sive, 2010; Huang et al., 2008). Although both species show F-actin accumulation in endodermal cells, the intestine of zebrafish mutants exhibits only a mildly reduced size without significant morphological changes (Huang et al., 2008). Interestingly, treatment with a Bmp inhibitor phenocopies the liver-loss phenotype but does not affect intestinal size in zebrafish, suggesting that mislocalisation of *bmp2a*-expressing LPM cells is primarily responsible for the liver phenotype. In medaka, the penetrance of the IA phenotype was incomplete (Table 2). Our data indicate that differential expression of Mypt family genes does not explain the intestinal specificity, implicating genetic background as a modifier of IA manifestation. These findings raise the possibility that genetic modifiers contribute to the variability and expressivity of the IA phenotype in medaka and may underlie interspecies differences. Supporting this, individuals with MYPT1 variants show highly variable clinical presentations (Contreras-Capetillo et al., 2024; Hughes et al., 2020; Picard et al., 2022), and IA has been reported in only a subset of cases. Notably, Picard et al. (2022) described a patient with IA at birth and PMDS, who remained otherwise healthy until age 29. This observation is consistent with our finding that a small number of medaka *mypt1* homozygous mutants can survive to adulthood, although this is rare. During the establishment of our mutant lines, we selectively bred individuals in which IA was frequently observed, and other developmental defects were relatively rare. This selective breeding likely enriched a genetic background predisposed to IA but less susceptible to other phenotypes. Given the more than 80 established laboratory medaka strains with diverse geographic and genetic origins (Katsumura et al., 2019), it would be interesting to cross our *mypt1* mutant with other genetic backgrounds to explore the influence of genetic modifiers on IA penetrance and to reveal additional phenotypes associated with *mypt1* disruption.

Mypt1 acts as a scaffold to provide a platform for assembly of the regulatory and catalytic subunits of the MLCP complex and for the recruitment of phosphatase substrates such as NMII. Conserved domains of Mypt1 that are vital for such molecular interaction are also found in medaka Mypt1. Our original *g1-4* mutant provides insight into one such domain: C2952A mutation introduces a premature stop codon after amino acids 983, thereby deleting a large part of the Prkg interacting domain of the WT 1078-amino acid protein (Fig. 1D). The LZ motif of this domain specifically mediates the interaction with Prkg1α (Surks et al., 1999). In vascular SM cells, activation of MLCP mediated by Prkg dephosphorylates pMrlc, which in turn induces vascular SM cell relaxation (Surks et al., 1999). Thus, loss of the LZ motif could explain increased contraction in the *g1-4* mutant. However, it remains to be clarified whether Prkg1α or other Prkg family members regulate intestinal epithelium integrity in developing intestine.

Our experiments using CRISPR-mediated knockout and morpholino knockdown of *myh11a* suggest potential involvement of SM-myosin in IA pathogenesis. Yet, SM-myosin expression was not detected in the intestinal region in which IA typically forms, suggesting that the SM layer is either absent or immature at the time of IA onset. *Myh11* encodes the SM-specific myosin heavy chain, a key component of the contractile machinery required for force generation and structural stability in SM cells (Deaton et al., 2023; Miano et al., 1994). However, the lack of fully developed SM cells at the timing of IA formation argues against a direct contribution of MYH11-mediated contraction of SM cells to the initial pathogenesis in IA (Fig. S14). In zebrafish, the *meltdown* (*mlt*) mutant – harbouring a constitutively active form of Myh11 – develops cystic expansion of the posterior intestine (Wallace et al., 2005b). This phenotype is thought to arise from Myh11-dependent stromal–epithelial signalling that disrupts epithelial architecture. A similar mechanism may operate in medaka *mypt1* mutants, in which loss of Mypt1 could enhance Myh11a activity. However, several distinctions suggest divergent downstream pathways: Snail family genes are upregulated in zebrafish *mlt* mutants but not in medaka; and whereas the *mlt* phenotype arises at 74 dpf – after SM encasement – the IA phenotype in medaka emerges by st. 28, before SM cells are identified surrounding the region in which IA develops (Gays et al., 2017; Wallace et al., 2005a). Thus, although Myh11a dysregulation and stromal–epithelial signalling may be involved in both models, their timing and molecular mediators differ. These considerations raise the possibility that Mypt1-dependent dysregulation of the actomyosin system in the intestine is not restricted to SM-myosin but may involve additional myosin isoforms expressed in earlier developmental stages. Consistent with this idea, blebbistatin treatment ameliorated the IA phenotype. Blebbistatin is known to inhibit multiple myosin isoforms at higher concentrations, which may account for its ability to rescue the phenotype (Limouze et al., 2004).

Interestingly, MYH11 also functions in contractile non-SM cells such as myofibroblasts, which can exert mechanical forces during development and repair (Pakshir et al., 2020). It is possible that *myh11a* functions in mesenchymal cells other than SM, contributing to mechanical stress in the developing gut and influencing epithelial morphogenesis. Although speculative, this possibility warrants further investigation. In addition, SM-specific *Mypt1* knockout (Mypt1^SMKO^) mice showed altered contractility but no apparent intestinal malformations or motility defects (He et al., 2013), further supporting the notion that canonical SM contraction is not directly responsible for IA pathogenesis. Together, these findings suggest that *myh11a* promotes IA through an SM-independent mechanism, potentially involving mechanically active mesenchymal populations such as myofibroblasts.

In addition to MYPT1 variants, ACTB variants have also been reported to cause IA (Fakhro et al., 2019; Saskin et al., 2017; Sibbin et al., 2021), highlighting the importance of tightly regulated actomyosin dynamics during intestinal development. Notably, variants of ACTB and ACTG1 are known to cause Baraitser-Winter syndrome 1 [BRWS1; Online Mendelian Inheritance in Man (OMIM) #243310] in humans, in which IA is an uncommon manifestation. This suggests that although disruption of actomyosin dynamics contributes to IA pathogenesis, it is not solely sufficient to induce the condition. Supporting this, pharmacological inhibition of NMII using blebbistatin completely rescued the IA phenotype in *mypt1* mutants, whereas treatment with calyculin A treatment – used to inhibit Pp1 and thus supress dephosphorylation of NMII – failed to phenocopy the IA phenotype. This discrepancy implies that Mypt1 may exert additional, non-canonical functions beyond its regulatory role in MLCP (Kiss et al., 2019), or that inhibition of Pp1 alone is insufficient to increase the amount of pNMII and contractility of intestinal epithelial cells. Identification of genetic modifiers that influence IA development in the *mypt1* mutant background may provide further mechanistic insight.

Our data show that ectopic accumulation of pMrlc and F-actin, likely indicative of actomyosin hypercontractility, occurs in a non-uniform pattern along the intestine in *mypt1* mutants. The cause of this spatial variability remains unknown. Notably, in a subset of embryos (two of 12; Fig. 7Eb), pMrlc levels were uniformly elevated throughout the intestine, raising the possibility that a stochastic or feedback-driven mechanism determines the eventual site of intestinal atresia. Moreover, we have not yet validated the molecular mechanism that underlies how hypercontractility in the intestinal epithelium disintegrates the epithelial layer structure. Further study is required to assess completely the mechanics underpinning embryonic epithelial integrity.

## MATERIALS AND METHODS

### Ethics statement and fish strains

All experiments were approved by the animal experimentation committee of Kyoto Prefectural University of Medicine (KPUM), licence numbers 27-123 and 2019-154. Fish were kept at 26°C on a 14 h light/10 h dark cycle in a constant re-circulating system of the in-house KPUM facility. The d-rR medaka strain, original *mypt1* mutant *g1-4* and hatching enzyme were obtained from National BioResource Project (NBRP; Okazaki, Japan). Embryos were staged according to the staging system of Iwamatsu (2004).

### Knock-in transgenic (KIT) medaka

To visualise the intestinal epithelium, we generated a KIT medaka line that expresses membrane-bound GFP (memGFP) under the control of the endogenous *foxa2* promotor (Fig. S2E), *Tg*[*foxa2:memGFP*]. *foxa2* is a well-known marker for the endoderm and its derived organs (Kobayashi et al., 2006). The targeting vector was constructed as described (Ansai and Kinoshita, 2017; Okuyama et al., 2013). This construct was integrated into the genome as previously described (Murakami et al., 2017). The sgRNA for the genomic target site was designed as previously described (Kimura et al., 2014).

### Detection of apoptosis by AO and TUNEL assay

To detect apoptotic cells, AO staining and TUNEL assays were performed. For AO staining, a stock solution of AO (0.05 mg/ml; Tokyo Chemical Industry, Tokyo, Japan) was prepared in distilled water and stored at 4°C in the dark. Prior to use, the stock solution was diluted to 17 μg/ml in hatching buffer (HB). Embryos at st. 27 were dechorionated and incubated in the AO solution for 30 min, then washed three times in fresh HB for 5 min each to remove excess dye. Fluorescent images were acquired using a DP70 digital camera mounted on an SZX16 stereomicroscope (Olympus, Tokyo, Japan). TUNEL assays were performed using the *In Situ* Apoptosis Detection Kit (Takara) according to the manufacturer's instructions. To visualise the intestinal epithelial membrane, embryos carrying the *Tg[foxa2:memGFP]* transgene were used. GFP signals were detected using a rabbit anti-GFP antibody (1:400; A6455, Invitrogen), followed by a goat anti-rabbit IgG antibody conjugated with Alexa Fluor 633 (1:400; Thermo Fisher Scientific).

### WISH

A *foxa2* probe was designed, and WISH was performed as described previously (Kobayashi et al., 2006; Takashima et al., 2007). Template DNAs for *snai1a* (EST clone number, olea55k06), *snai1b* (olea23g07) and *snai2* (olsp48a08) were obtained from NBRP Medaka.

### Histology

Embryos fixed with 4% paraformaldehyde (PFA) were embedded in paraffin or resin (Technovit 7100, Heraeus), and 6 µm tissue sections were prepared. Paraffin sections were stained with Haematoxylin and Eosin, while plastic sections were stained with Haematoxylin only, followed by analysis using a BX51 microscope (Olympus).

### Immunohistology

For staining SM-myosin, laminin and cytokeratin, 80% methanol/20% DMSO (Dent's solution) was used for fixation at −20°C. For simultaneous staining of laminin or E-cadherin with phalloidin, embryos were fixed in 1.5% PFA in 1.5× PBS containing 0.1% Tween 20 at room temperature (RT) for 1 h, and then transferred to 4°C. In other cases, embryos were fixed in 4% PFA in 1.5× PBS containing 0.1% Tween 20 at RT. If Dent's fixation was used, embryos were de-chorionated with medaka hatching enzyme before fixation. Then, non-specific antibody binding sites were blocked with 1% DMSO, 2% bovine serum albumin, 10% normal goat serum, 0.1% Triton X-100 in 1× PBS. Embryos fixed in PFA were permeabilised by incubation in 1× PBS containing 2% Triton X-100 for at least 2 h before blocking of non-specific binding sites. The following commercially available antibodies were used: anti-GFP (1:2000; ab13970, lot GR3190550-29, Abcam), anti-cytokeratin (1:100; clone AE1/AE3, ab27988, Abcam), anti-SM-myosin (Myh11; 1:50; BT562, lot YS171691, Biomedical Technologies), anti-laminin (1:100; L9393, lot 104M4759V, Sigma-Aldrich), anti-phosphorylated myosin light chain 2 (Ser19) (1:20; 3671, lot 7, Cell Signaling Technology), anti-PH3 (Ser10) (1:500; 06-570, lot 2465253, Millipore), anti-Pkcζ (1:800; ab5964, lot GR79724-12, Abcam) and anti-E-cadherin (1:50; 610181, lot 5065795, BD Biosciences). Alexa Fluor-633-Phalloidin (1:20; A22284, lot 2110449, Thermo Fisher Scientific) was used to mark F-actin. Detection of primary antibodies was performed using Alexa Fluor-488, -555 goat anti-rabbit, chicken or mouse IgG (1:400; Invitrogen). Images were acquired with an Olympus FV1200 confocal microscope or Zeiss LSM900, and *yz*-planes were reconstructed using FIJI (Schindelin et al., 2012). Abnormal accumulation of pMrlc or F-actin was judged based on (1) obviously higher immunofluorescence signal in the middle part of the intestine compared with other areas, and (2) obviously higher immunofluorescence signal at apical and also basal areas of the intestinal epithelium where strong signal was never observed in the WT. Representative single-plane confocal images (not *z*-projections) are shown in the figures.

### CRISPR/Cas9-mediated mutagenesis of *mypt1*

The sgRNA targeting exon 1 of *mypt1* was designed using the web tool ‘Search for CRISPR target site with micro-homology sequences’, with the parameter ‘micro-homology sequences’ set to four bases. sgRNA synthesis was performed as previously described (Ansai and Kinoshita, 2014). The target sequence is shown in Fig. S1A. A mixture containing 150 ng/µl sgRNA and 200 ng/µl Cas9 mRNA was microinjected into one- to two-cell-stage embryos. Injected embryos were raised to adulthood, and eight germline-transmitting F0 founders were identified by genotyping pooled F1 offspring. One of these founders, #4-6, was outcrossed to WT fish, and the resulting F1 progeny were genotyped. F1 fish harbouring the mutation (Fig. 1; Fig. S2) were used for subsequent analyses.

### CRISPR/Cas9 knockout of *myh11a*

To knock out *myh11a*, three overlapping sgRNAs targeting exon 5 – which encodes part of the ATP-binding domain – were designed using the CRISPR-Cas9 guide RNA design checker [Integrated DNA Technologies (IDT)]. Off-target effects were evaluated via BLAST search against the medaka genome using the Ensembl genome browser. The overlapping design of sgRNAs was intended to increase mutagenesis efficiency in the targeted region (Fig. S13A). Each sgRNA was prepared following the manufacturer's protocol (IDT). A mixture containing 1.0 µg/µl Alt-R™ S.p. Cas9 Nuclease V3 (IDT) and 5.0 µM of each sgRNA, diluted in RNase-free water was injected into one- to two-cell-stage medaka embryos.

### Morpholino knockdown of *myh11a*

Antisense Morpholino (Gene Tools) targeting the splice donor site of exon 2of medaka *myh11a* was used for knockdown (Fig. S4E). The Standard Control Oligo supplied by Gene Tools was used as a negative control.

### Genotyping

WT and mutant *mypt1* alleles were distinguished using a PCR-restriction fragment length polymorphism method. Genomic DNA from lysed tissue was subjected to PCR using Quick Taq^®^ HS DyeMix Taq polymerase (TOYOBO), with an initial denaturation at 94°C for 2 min, followed by 45 cycles of 94°C denaturation for 30 s, annealing and extension at 68°C for 50 s. The primers used are shown in Tables S1 and S2. PCR products were digested with the restriction enzyme *Hae*III (TOYOBO), and analysed by electrophoresis in 3% agarose gels (02468-95, Nacalai) in 1× TAE buffer [40 mM Tris(hydroxymethyl)aminomethane (Nacalai Tesque), 20 mM acetic acid (FUJIFILM Wako Pure Chemical) and 1 mM EDTA (Nacalai Tesque); pH was adjusted to 8.0].

### Chemical inhibition of actomyosin contractility

To examine the effect of NMII inhibition on IA development, st. 25 embryos were treated with either 0.1% DMSO (control) or 50 µM (−)-blebbistatin (021-17041, FUJIFILM), dissolved in 0.1% DMSO. After a 2-h incubation, embryos were thoroughly washed and raised until 4 dpf for phenotypic analysis. Furthermore, embryos were raised to 9 dpf for extended observation, imaging and genotyping. To assess the effects of increased myosin phosphatase inhibition, WT embryos were treated from st. 23 to st. 32 with either 0.1% DMSO (control) or 0.1 µM calyculin A (101932-71-2, FUJIFILM), also dissolved in 0.1% DMSO. Phenotypic evaluation was performed after the treatment, and embryos were further raised to 9 dpf for evaluation.

### Phylogenetic analysis

Multiple sequence alignments were performed using CLUSTALW with default parameters, followed by phylogenetic tree construction using the PhyML-bootstrap method (Kyoto University Bioinformatics Center). The protein sequences used for the alignment are listed in Table 3.

### RT-PCR

Total RNA was extracted using TRIzol reagent (Invitrogen) according to the manufacturer's protocol. For small tissue samples, RNA was purified using a Direct-zol RNA Kit (Zymo Research). Reverse transcription and PCR were carried out using superscript III (Invitrogen) and Quick Taq^®^ HS DyeMix (TOYOBO), respectively, following the manufacturer's instructions. Molecular mass marker (DM2100; SMOBIO) was used as a standard.

### Statistical analysis

Statistical analyses were performed using EZR (Kanda, 2013).

### Use of artificial intelligence tools

ChatGPT (OpenAI) was used for English language editing during the initial drafting of the manuscript.

## Supplementary Material

10.1242/dmm.052605_sup1 Supplementary information
