# Supplementary material for: A genetic model of congenital intestinal atresia implicates Mypt1 in epithelial organisation
Source: Dis Model Mech. 2026 Mar 10;19(2):dmm052605. doi: 10.1242/dmm.052605 (PMC13035062; doi:10.1242/dmm.052605)
Supplement: Supplementary information [file dmm-19-052605-s1.pdf]

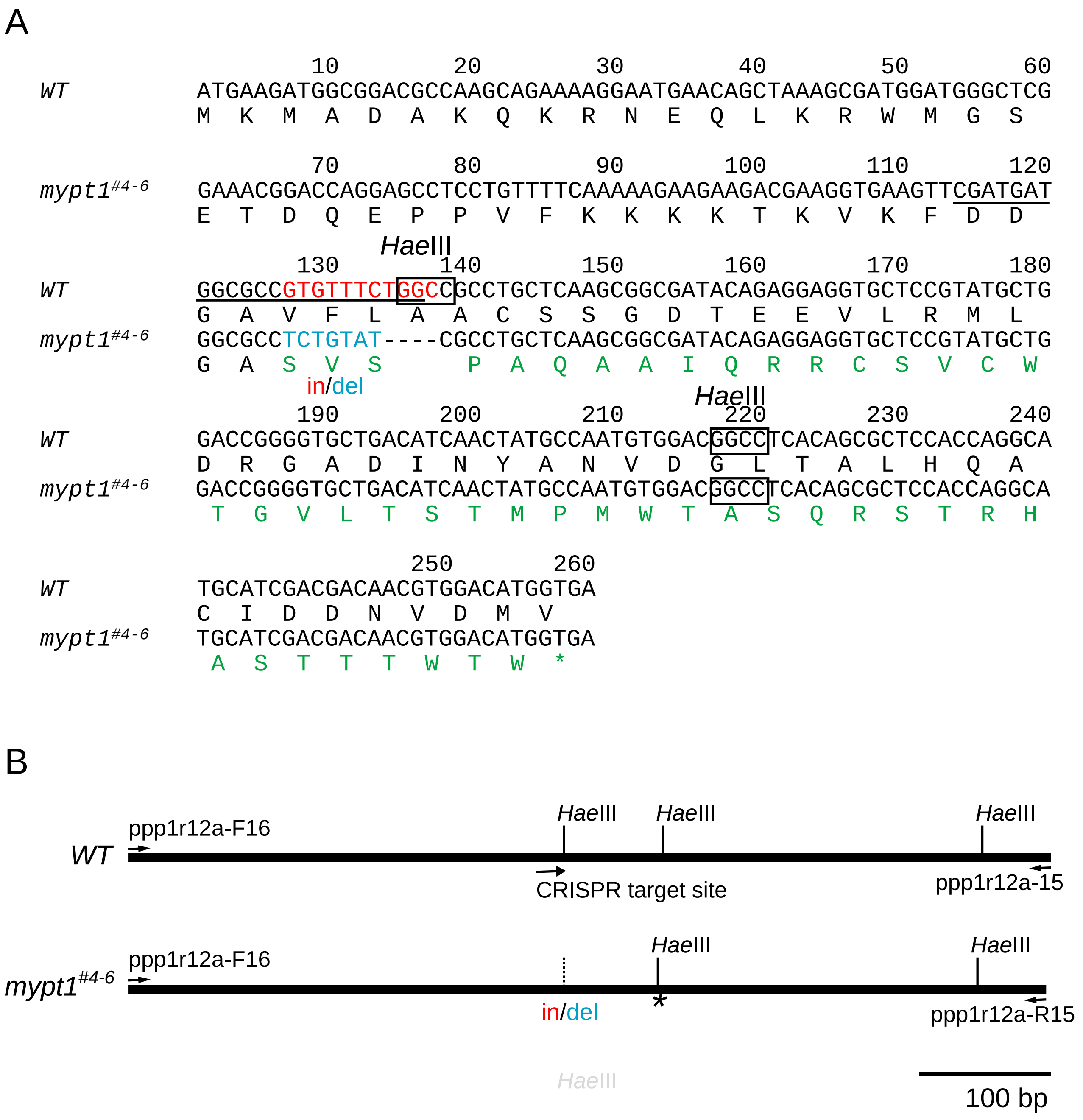

Fig. S1. Comparison of *WT* and *mypt1*$^{\#4\text{-}6}$ allele sequences. (A) The 11-nucleotide deletion and 7 nucleotide insertion are shown in red and blue, respectively. The CRISPR target site is underlined. The predicted amino acid sequence resulting from the frame shift is shown in green. Asterisk indicates premature stop codon. *Hae*III sites (GGCC) that are used for genotyping by restriction fragment length polymorphism (RFLP) assays are indicated. (B)

Disease Models & Mechanisms • Supplementary information

Schematic outline of PCR products amplified from *WT* and *mypt1*$^{\#4\text{-}6}$ alleles. Primer positions (*ppp1r12a*-F15 and *ppp1r12a*-R16), the CRISPR target site and *Hae*III sites are indicated. The *Hae*III site lost in *mypt1*$^{\#4\text{-}6}$ is shown in grey. The introduced stop codon in *mypt1*$^{\#4\text{-}6}$ is marked by an asterisk.

Disease Models & Mechanisms • Supplementary information

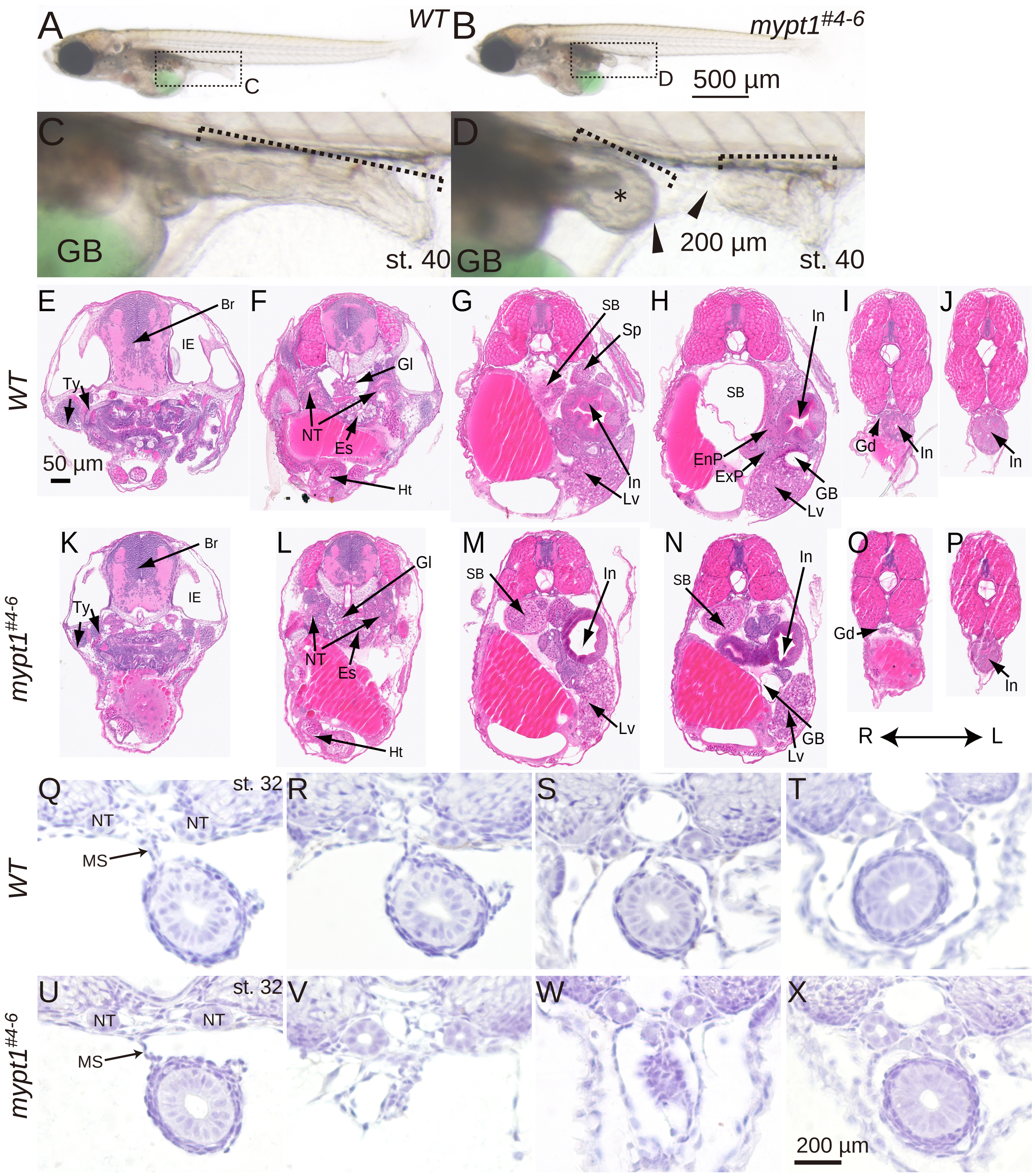
A
WT
C
B
mypt1#4-6
D
500 µm
C
GB
st. 40
D
*
200 µm
GB
st. 40
E
Br
IE
Ty
50 µm
F
Gl
NT
Es
Ht
G
SB
Sp
In
Lv
H
SB
In
EnP
ExP
GB
Lv
I
Gd
In
J
In
WT
K
Br
IE
Ty
L
Gl
NT
Es
Ht
M
SB
In
Lv
N
SB
In
GB
Lv
O
Gd
P
In
R
L
mypt1#4-6
Q
st. 32
NT
NT
MS
R
S
T
WT
U
st. 32
NT
NT
MS
V
W
X
200 µm
mypt1#4-6

Fig. S2. Organogenesis proceeds normally in *mypt1* mutants except for the presence of IA. (A–D) Dilation of the intestine in *mypt1* mutants. Ventral views of *WT* (A, C) and *mypt1#4-6* (B, D) embryos at st. 40 in the Tg[*foxa2:memGFP*] medaka line. GFP fluorescence and brightfield images are shown in (A, B) and (C, D), respectively. White dotted lines outline the intestine. Note the significant dilation anterior to the IA lesion in the mutant (asterisk). LV, liver. (E–P) Histological sections of st. 40 embryos. *WT*: (E–J); *mypt1* mutant: (K–P). Panels show comparable anatomical levels: (E and K), (F and L), (G and M), (H and N), (I and O), and (J and P). Abbreviations: EnP, endocrine pancreas; Es, esophagus; ExP, exocrine pancreas; GB, gall bladder; Gd, gonad; Gl, glomerulus; Ht, heart; IE, inner ear; In, intestine; Lv, liver; NT, nephric tubule; Sp, spleen; AB, swim bladder; Ty, thyroid; R, right; L, left. (Q–X) Histological micrographs of the intestine in *WT* (Q–T) and *mypt1*$^{\#4\text{-}6}$ (U–X) embryos at st. 32, stained with haematoxylin. (U) Region anterior to IA; (V) IA lesion; (W) anterior blind-end of posterior intestine; (X) region posterior to the IA. *WT* sections (Q–T) correspond anatomically to (U–X). Note normal morphology in (U) and (X). MS, mesentery; NT, nephric tubule. The timing of swim bladder inflation varies among individuals at the hatching stage, including in WT embryos. Accordingly, the wild-type embryo shown in (H) and the *mypt1*$^{\#4\text{-}6}$ mutants shown in (M, N) may reflect individuals at slightly different stages with respect to swim bladder swim bladder inflation.

Disease Models & Mechanisms • Supplementary information

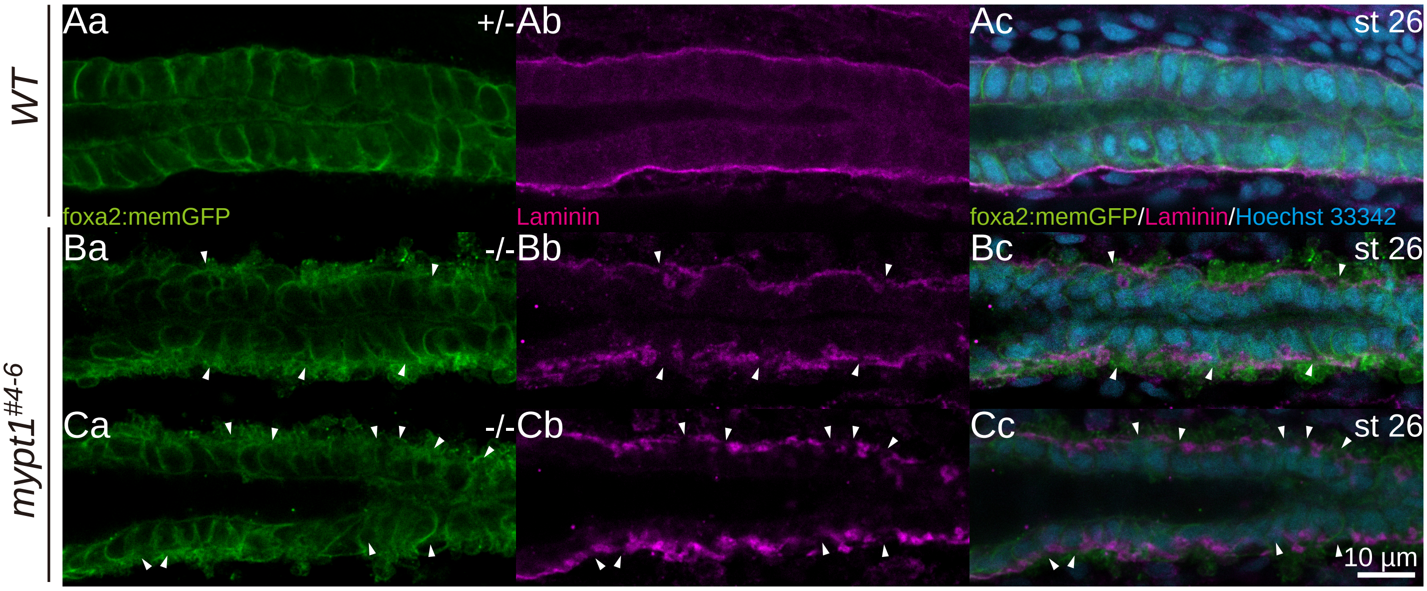

Fig. S3. Basement membrane fragmentation in the mutant. memGFP (Aa, Ba, Ca) and laminin (Ab, Bb, Cb) staining in *WT* (A) and *mypt1*$^{\#4\text{-}6}$ (B, C) as left lateral view at st. 26. Arrowheads indicate discontinuous laminin staining. Scale bar: 10 µm.

Disease Models & Mechanisms • Supplementary information

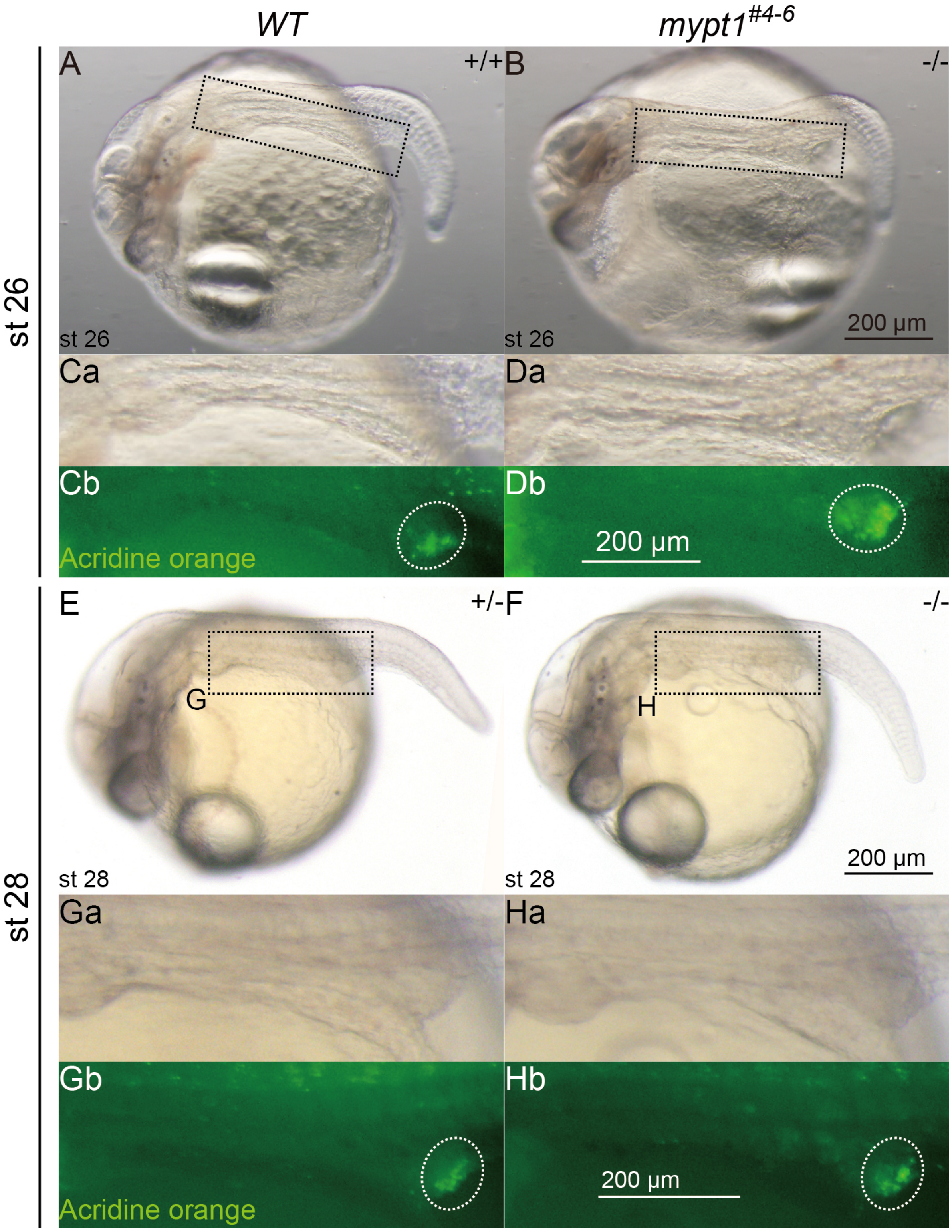

Fig. S4. Acridine orange staining at stages 26 and 28. (A–D) Acridine orange staining of *WT* (Cb, n = 7) and *mypt1*$^{\#4\text{-}6}$ (Db, n = 7) embryos at stage 26. (E–H) Acridine orange staining of WT (Gb, n = 11) and *mypt1*$^{\#4\text{-}6}$ (Hb, n = 11) embryos at stage 28. Dotted boxes in (A, B, E, F) indicate regions enlarged in (C, D, G, H), respectively. (Ca, Da, Ga, Ha) Brightfield images corresponding to Cb, Db, Gb, and Hb, respectively. Dotted ellipses mark apoptotic cells surrounding the cloacal opening, where apoptosis is normally observed (Parkin et al., 2009).

Disease Models & Mechanisms • Supplementary information

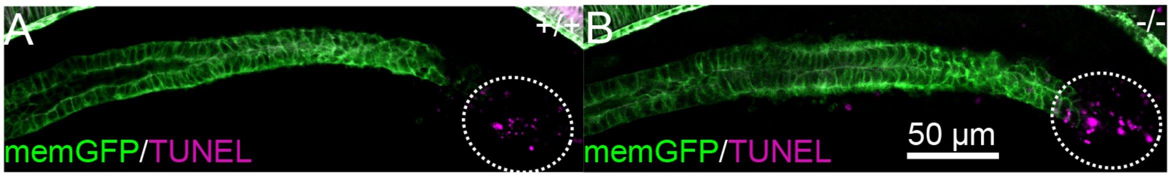

Fig. S5. Cell death in the intestine is not significantly affected 1 in *mypt1*$^{\#4\text{-}6}$ mutants. (A, B) TUNEL assays of *WT* (A) and mypt1 mutant (B) embryos at st. 26. Dotted ellipses indicate a number of apoptotic cells surrounding the cloaca opening where apoptosis is always observed (Parkin et al., 2009). Scale bar, 50 μm.

Disease Models & Mechanisms • Supplementary information

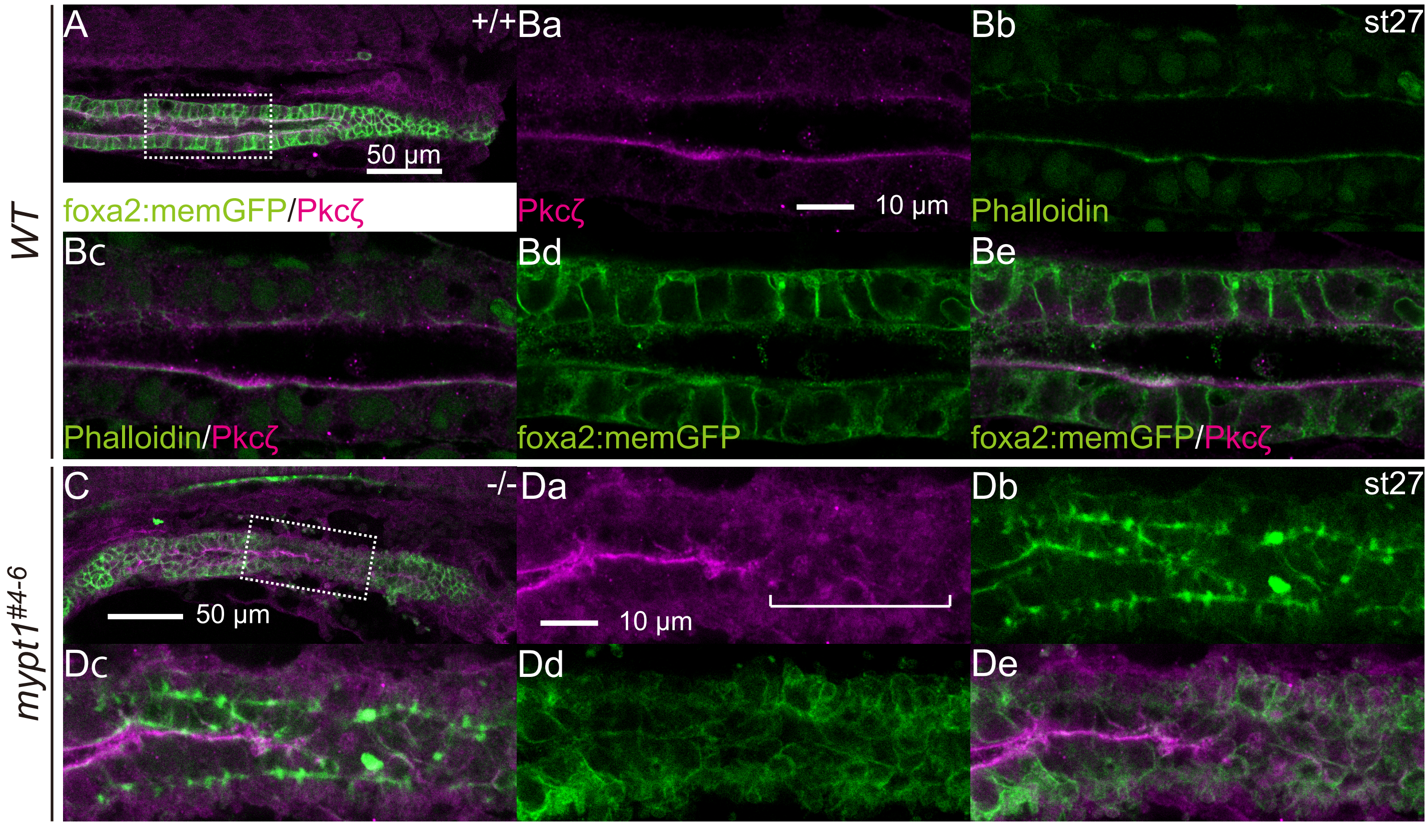

Fig. S6. PKCζ localization in *mypt1*$^{\#4\text{-}6}$ mutant embryos at stage 27. (A) WT and (B) *mypt1*$^{\#4\text{-}6}$ embryos. Panels A and B correspond to Fig. 2J and 2K, respectively, in the main text and are shown here for reference. Dotted boxes in (Aa) and (Ba) indicate regions enlarged in (Ab-Af) and (Bb-Bf), respectively. In *WT* embryos, PKCζ is localised to the apical domain at stage 27 (Bb). In *mypt1*$^{\#4\text{-}6}$ embryos, apical PKCζ localization is partially lost (Bb, white brackets). Corresponding phalloidin (Ac, Bc), memGFP (Ae, Be), and merged images are shown in (Ad, Af, Bd, Bf), respectively.

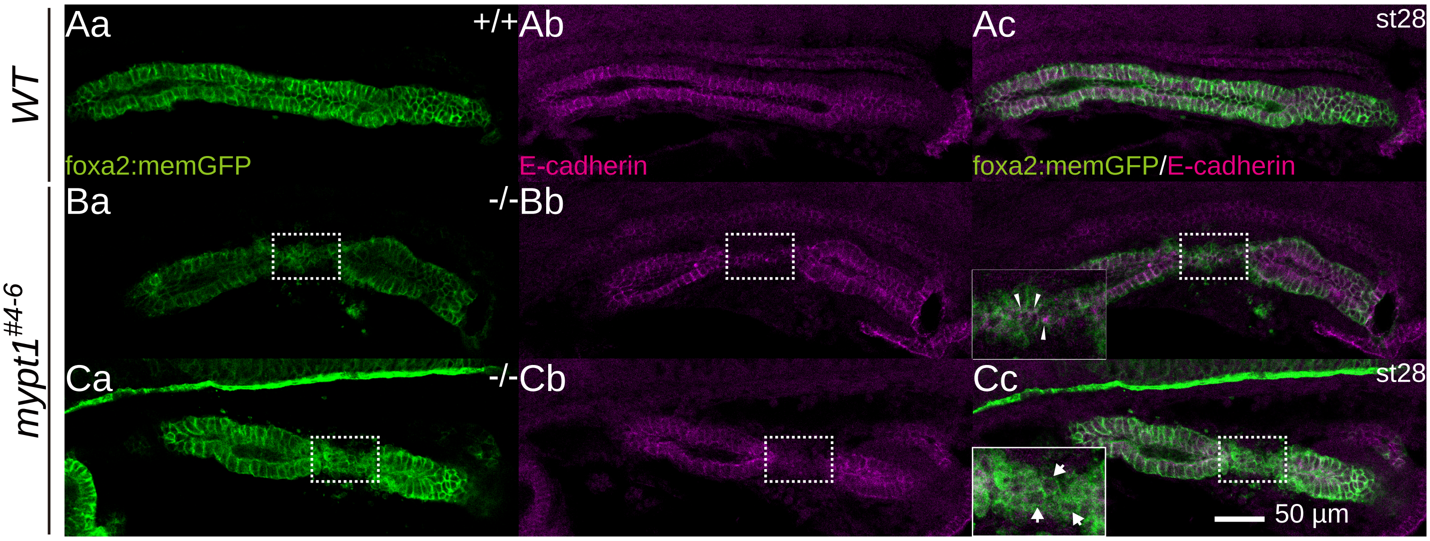

Fig. S7. E-cadherin localisation is reduced or lost in the degrading epithelium. (A) *WT* and (B, C) *mypt1*$^{\#4\text{-}6}$ embryos. Dotted boxes in (B, C) indicate regions enlarged in the insets. Arrowheads indicate E-cadherin localisation in regions undergoing epithelial degradation and intestinal atresia (B). Arrows indicate loss of E-cadherin signals in regions undergoing epithelial degradation and intestinal atresia (C).

Disease Models & Mechanisms • Supplementary information

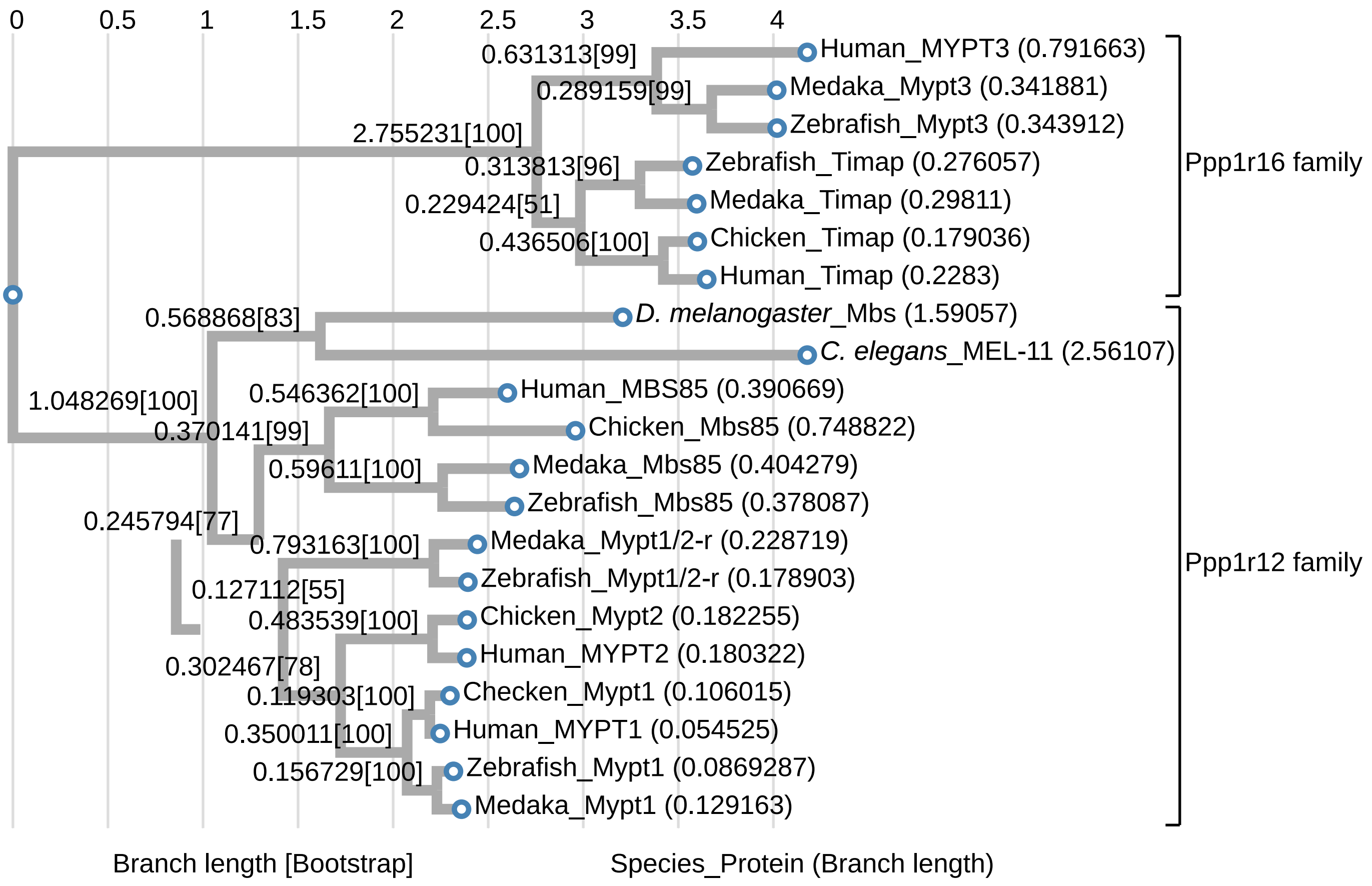

Fig. S8. Phylogenetic analysis of the Mypt protein family. Phylogenetic tree conducted from protein sequences of *mypt* family genes listed in Table 3.

Disease Models & Mechanisms • Supplementary information

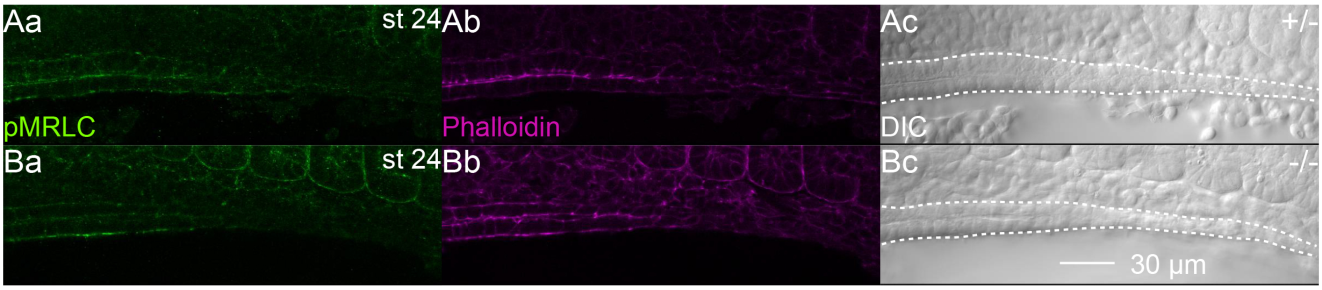

Fig. S9. Actomyosin is not activated in the developing intestine of *mypt1*$^{\#4\text{-}6}$ mutants at st. 24. (Aa, Ba) Fluorescence micrographs of pMRLC in WT (Aa) and *mypt1*$^{\#4\text{-}6}$ embryos (Ba). (Ab, Bb) Fluorescence micrographs of F-actin stained with phalloidin in WT (Ab) and *mypt1*$^{\#4\text{-}6}$ embryos (Bb). No significant accumulation of pMRLC (Ba) or F-actin (Bb) is observed in the intestinal epithelium of *mypt1*$^{\#4\text{-}6}$ mutants. (Ac, Bc) Differential interference contrast images corresponding to (Aa, Ab) and (Ba, Bb), respectively. White dotted lines outline the intestine. Scale bar, 30 µm.

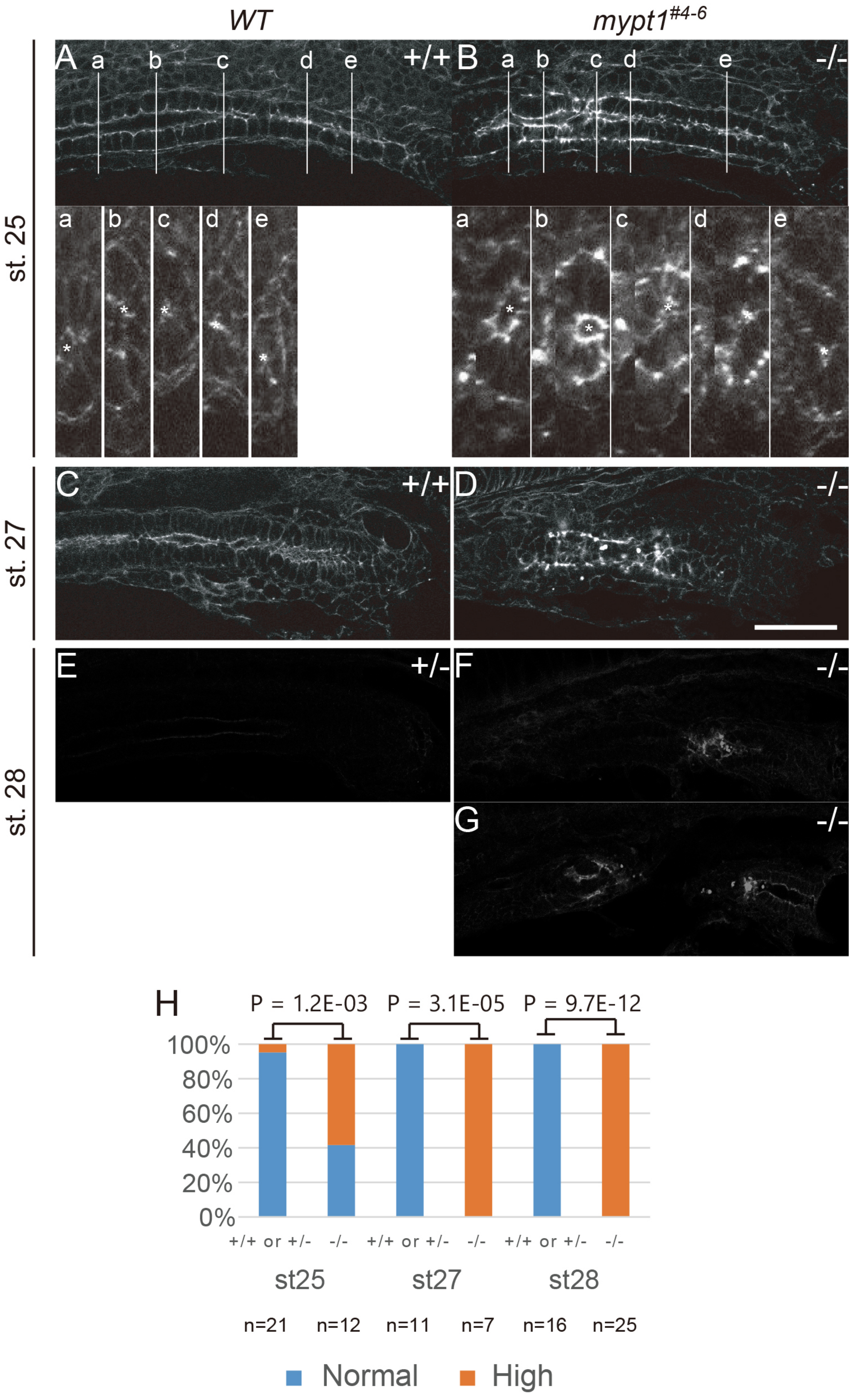

Disease Models & Mechanisms • Supplementary information

Fig. S10. Abnormal accumulation of F-actin in the developing intestine of *mypt1* mutant embryos. (A–G) Fluorescence micrographs of F-actin in *WT* (A, C, E) and *mypt1*$^{\#4\text{-}6}$ (B, D, F, G). Embryos at st. 25 are shown in (A, B), embryos at st. 27 in (C, D), and embryos at st. 28 in (E–G). Notable accumulation of F-actin is observed in the intestinal epithelium of mutant embryos (Bb, Db, Eb, Gb). (a–e) Short-axis sections of the intestine. Asterisks indicate the intestinal lumen. (H) Quantification of embryos exhibiting abnormally increased F-actin signals in the embryonic intestine (Fisher's exact test).

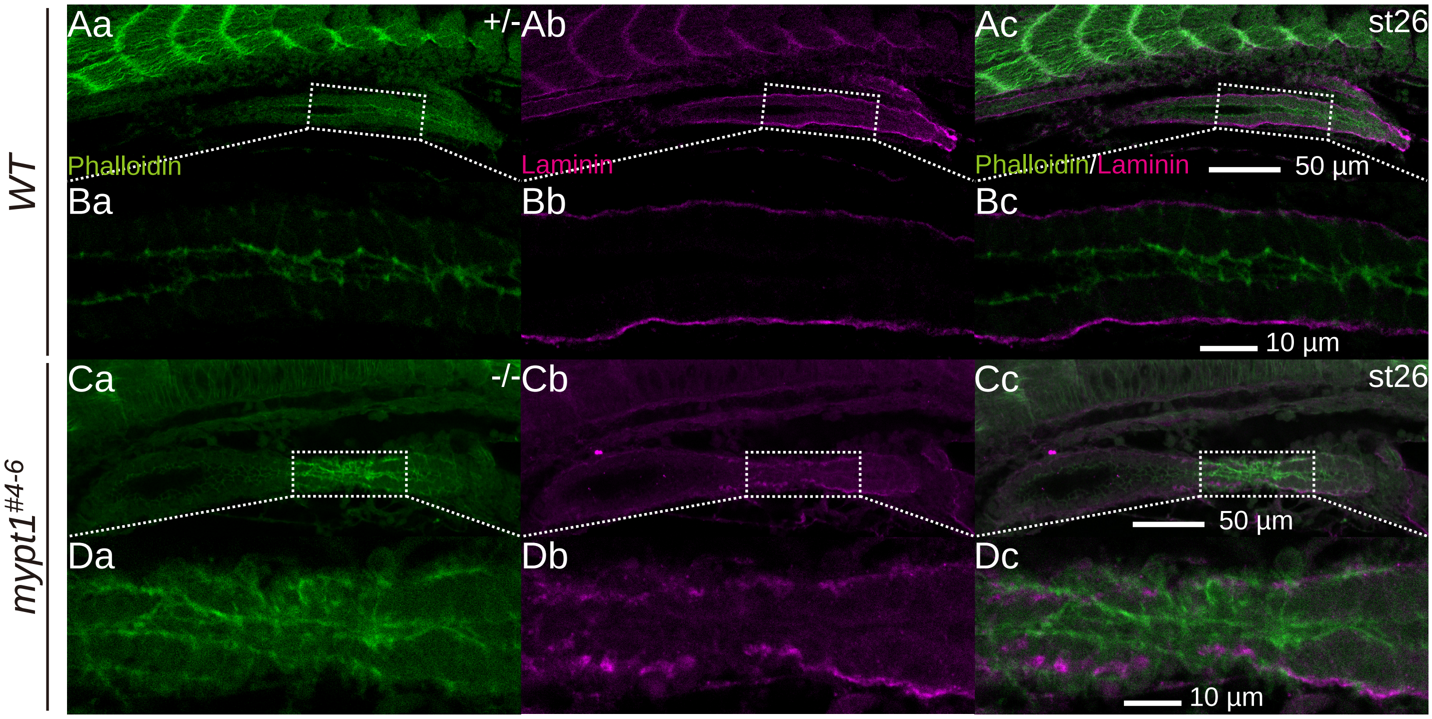

Fig. S11. Basement membrane fragmentation overlaps with regions showing elevated F-actin signals. Phalloidin (Aa, Ba, Ca, Da) and laminin (Ab, Bb, Cb, Db) staining in *WT* (A, B) and *mypt1*$^{\#4\text{-}6}$ (C, D), shown as left lateral views. White dotted boxes in (A) and (C) indicate regions magnified in (B) and (D), respectively. Magnified views highlight regions where basement membrane fragmentation coincides with elevated F-actin levels in *mypt1* mutant embryos. Scale bars: 50 µm in (A, C); 10 µm in (B, D).

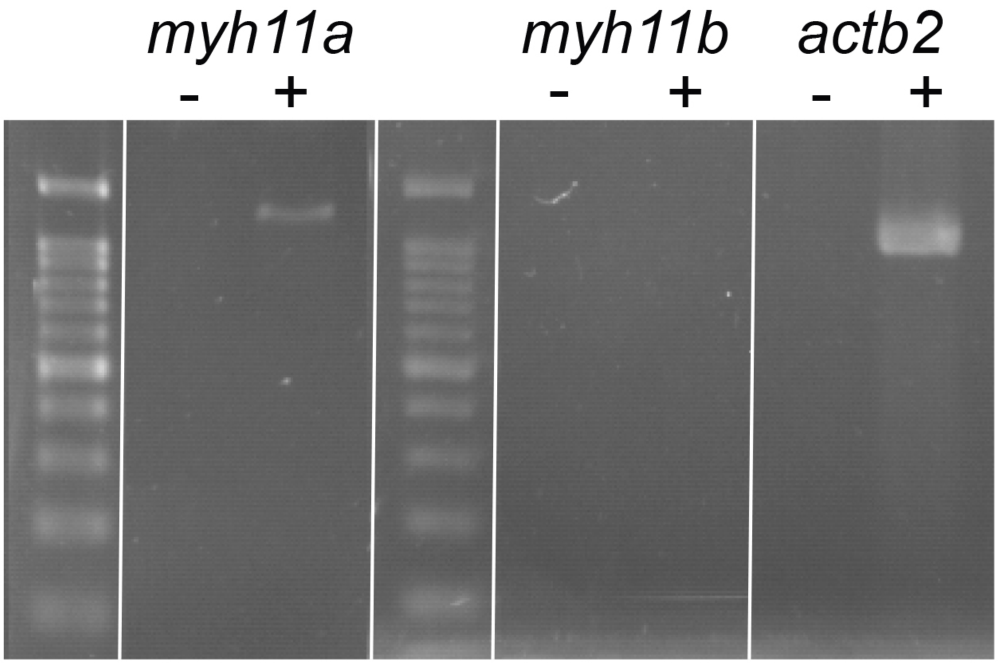

Fig. S12. RT-PCR analysis of *myh11a* and *myh11b*. PCR was performed with (+) or without (−) first-strand complementary DNA synthesized from 2 dpf embryos. Primers used for PCR are listed in Table S2. MW, molecular weight marker (DM2100, SMOBIO).

Disease Models & Mechanisms • Supplementary information

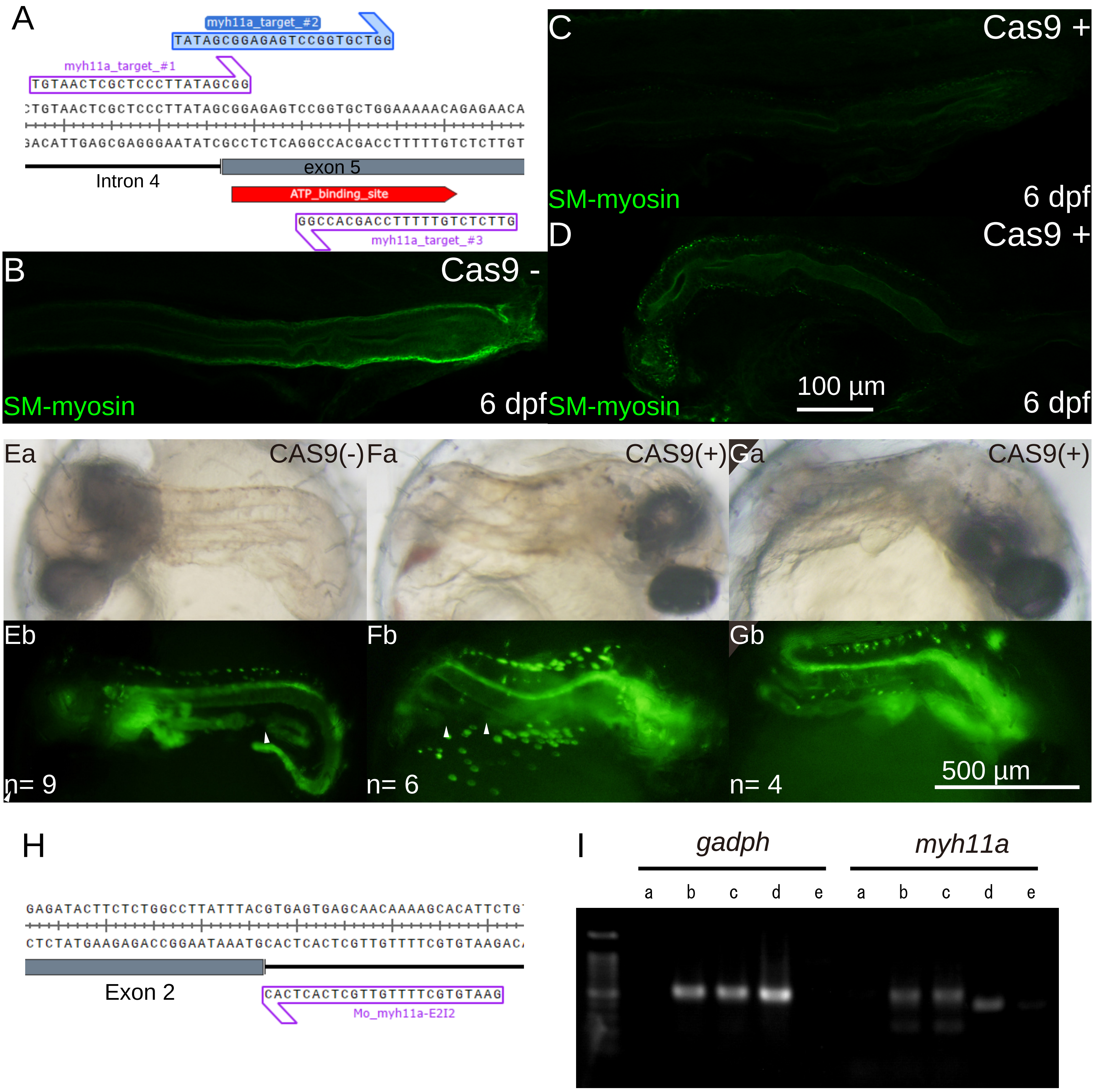

a1: - template
b,c: Mo-*myh11a*-E2I2
d: Mo uninjected
e: WT genome

Disease Models & Mechanisms • Supplementary information

Fig. S13. ***myh11a*** **knockdown partially rescued ntestinal atresia. (A) CRISPR target sites** within the *myh11a* gene. (B–D) Expression of SM-myosin in 6 dpf embryos, into which *myh11a* sgRNA was injected at 1-2 cell stage: without Cas9 nuclease (B); with Cas9 (C and D). Scale bar: 100 µm. (E-G) Whole embryo images of SM-myosin knockdown embryos. The bright field (Ea, Fa, Ga) and fluorescent images (Eb, Eb, Eb) of 4 dpf embryos without Cas9 nuclease (E); with Cas9 accompanied with IA (F) and without IA (G). Arrowheads indicate the blind ends of atretic region. Scale bar: 500 µm.

(H) Morpholino target site within *myh11a*. (I) RT-PCR analysis of *myh11a* morpholino-injected embryos. Lane 1: control; lanes 2 and 3: morpholino-injected; lane 4: *WT* cDNA; lane 5: *WT* genomic DNA.

Disease Models & Mechanisms • Supplementary information

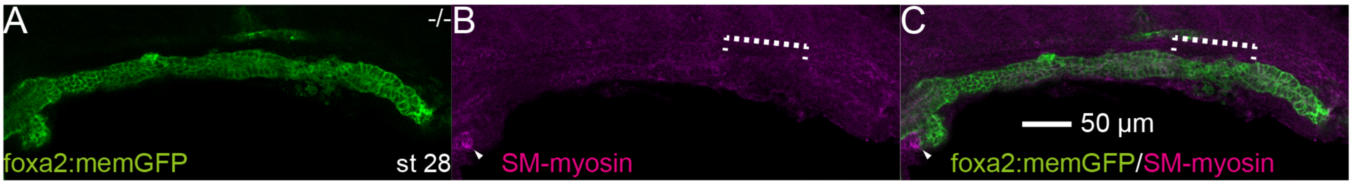

Fig. S14. No obvious SM layer is observed surrounding the intestinal epithelium in the *mypt1*$^{\#4\text{-}6}$ mutant at st. 28. (A) GFP signal marking the intestinal epithelium. (B) SM-myosin immunofluorescence. (C) Merged image. Brackets indicate the region where IA is developing. Arrowheads indicate SM-myosin expression surrounding the developing air bladder. Scale bar, 50 µm.

Table S1. Sequences of PCR primers used in Figs 6 and S13 and related to Fig. 5.

gene	Forward	Reverse
mypt1	TCCTCCACTTTCCTCATATCTGCTGATGG	TGGCCAAAAATCGTCAGGTGTGTTCG
myh11a	ATGTCTAAGAAGGCCCCGAGTG	GGAACTTCATGCCGTTTCTTCC
dagph	GTATCAATGGTTTTGGCCGTATC	AGTGATGGCATGAACTGTGCTC
actb2	GATGCCCCTCGTGCTGTCTTTCC	CCGTCAGGATCTTCATGAGG
mypt1/2-r	GACGGCAGGATCAGTTGCAG	CTCCTCTTTTCGGGCCTTGTC
mbs85	CGCCACCGTATGTCTTGTTT	CACCGTGATCCAAGAGGAAC
snail1a	CACTAAAGCTATCACTCCGTTGCT	GGTCCCAGCCTTCTTATGGTTC
snail1b	CGCGTCTTCCAACATTTACGCA	ACCATACACAGACAGCTGTCTCA
snail2	GGAGTTTGTTCTTTACGCAGCGAA	AGCTGGCAAACTGTTTCTACAGG

Table S2. Sequences of PCR primers used in Fig. S12.

gene	Forward	Reverse
myh11a	GAAACATGATGCAAGATCGTGAGG	AGAGGGTCCATGTTCTTTGTGAGC
myh11b	GAGAAGGAGAGGAACAACGAGCAG	GTACAGATTGGGGTCCAGATCCAG
actb2	GATGCCCCTCGTGCTGTCTTTCC	GAGCAGGACAGGGTGCTCCTCA

Disease Models & Mechanisms • Supplementary information

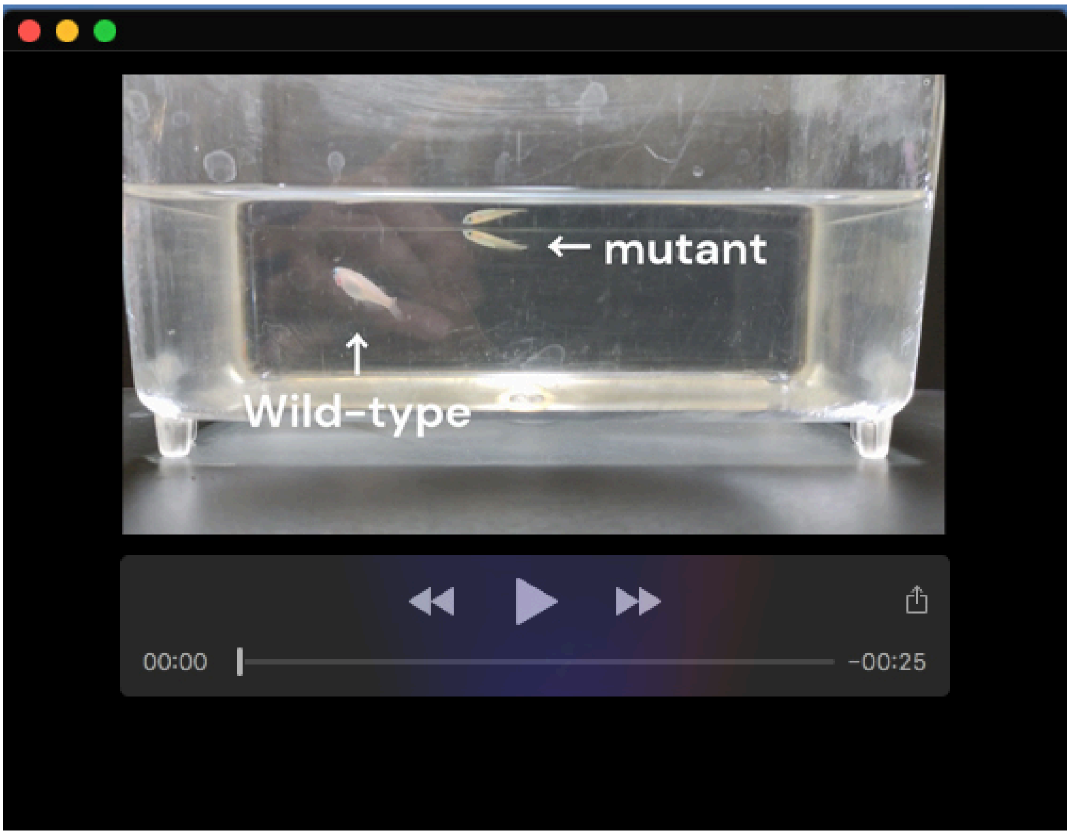

Movie 1. Unhealthy mutant.

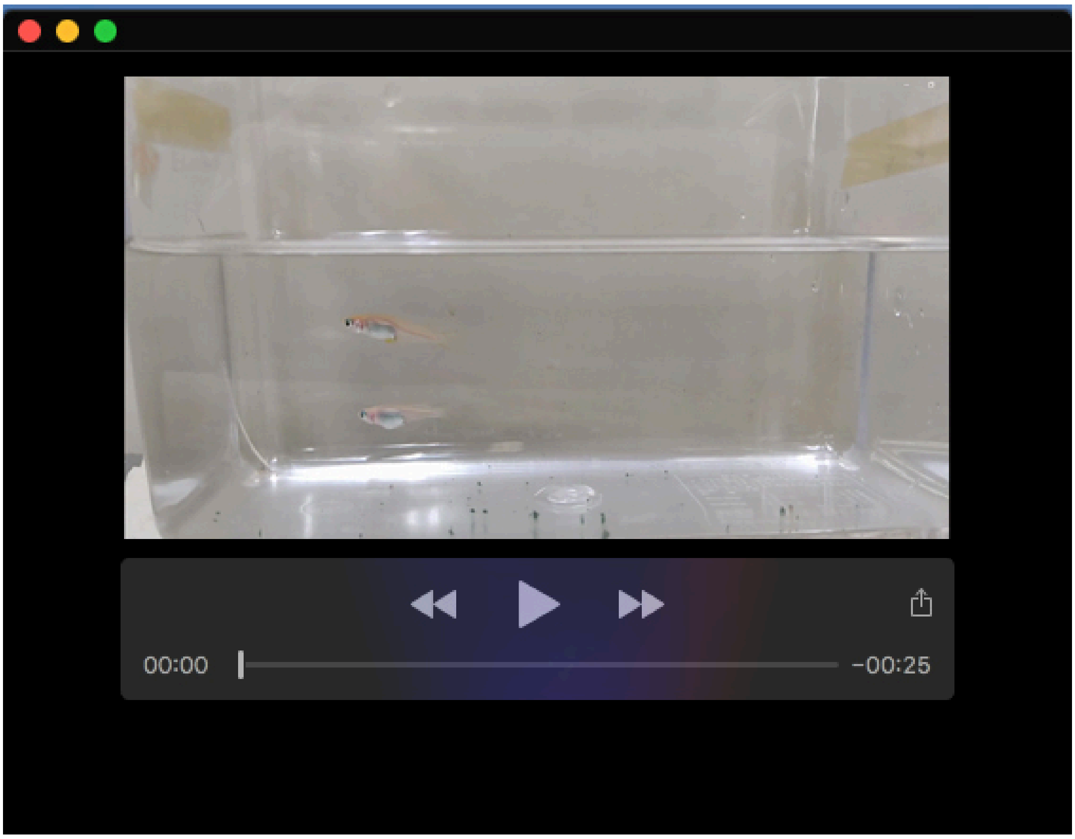

Movie 2. Apparently normal mutant.

Disease Models & Mechanisms • Supplementary information
